# Dual BTK/SYK inhibition with CG-806 (luxeptinib) disrupts B-cell receptor and Bcl-2 signaling networks in mantle cell lymphoma

**DOI:** 10.1038/s41419-022-04684-1

**Published:** 2022-03-16

**Authors:** Elana Thieme, Tingting Liu, Nur Bruss, Carly Roleder, Vi Lam, Xiaoguang Wang, Tamilla Nechiporuk, Geoffrey Shouse, Olga V. Danilova, Daniel Bottomly, Shannon K. McWeeney, Jeffrey W. Tyner, Stephen E. Kurtz, Alexey V. Danilov

**Affiliations:** 1 City of Hope National Medical Center, Duarte, CA USA; 2 Knight Cancer Institute, Oregon Health and Science University, Portland, OR USA; 3 Division of Hematology & Medical Oncology, Oregon Health & Science University, Portland, OR USA; 4 Division of Bioinformatics and Computational Biology, Department of Medical Informatics and Clinical Epidemiology, Oregon Health and Science University, Portland, OR USA; 5 Oregon Clinical and Translational Research Institute, Portland, OR USA; 6 Department of Cell, Developmental & Cancer Biology, Oregon Health & Science University, Portland, OR USA

**Keywords:** Target validation, B-cell lymphoma

## Abstract

Aberrant B-cell receptor (BCR) signaling is a key driver in lymphoid malignancies. Bruton tyrosine kinase (BTK) inhibitors that disrupt BCR signaling have received regulatory approvals in therapy of mantle cell lymphoma (MCL). However, responses are incomplete and patients who experience BTK inhibitor therapy failure have dire outcomes. CG-806 (luxeptinib) is a dual BTK/SYK inhibitor in clinical development in hematologic malignancies. Here we investigated the pre-clinical activity of CG-806 in MCL. In vitro treatment with CG-806 thwarted survival of MCL cell lines and patient-derived MCL cells in a dose-dependent manner. CG-806 blocked BTK and SYK activation and abrogated BCR signaling. Contrary to ibrutinib, CG-806 downmodulated the anti-apoptotic proteins Mcl-1 and Bcl-xL, abrogated survival of ibrutinib-resistant MCL cell lines, and partially reversed the pro-survival effects of stromal microenvironment-mimicking conditions in primary MCL cells. Dual BTK/SYK inhibition led to mitochondrial membrane depolarization accompanied by mitophagy and metabolic reprogramming toward glycolysis. In vivo studies of CG-806 demonstrated improved survival in one of the two tested aggressive MCL PDX models. While suppression of the anti-apoptotic Bcl-2 family proteins and NFκB signaling correlated with in vivo drug sensitivity, OxPhos and MYC transcriptional programs were upregulated in the resistant model following treatment with CG-806. *BAX* and *NFKBIA* were implicated in susceptibility to CG-806 in a whole-genome CRISPR-Cas9 library screen (in a diffuse large B-cell lymphoma cell line). A high-throughput in vitro functional drug screen demonstrated synergy between CG-806 and Bcl-2 inhibitors. In sum, dual BTK/SYK inhibitor CG-806 disrupts BCR signaling and induces metabolic reprogramming and apoptosis in MCL. The Bcl-2 network is a key mediator of sensitivity to CG-806 and combined targeting of Bcl-2 demonstrates synergy with CG-806 warranting continued exploration in lymphoid malignancies.

## Introduction

Mantle cell lymphoma (MCL) is a rare subtype of non-Hodgkin’s lymphoma (NHL) manifesting rearrangement of cyclin D1. MCL is characterized by significant genetic heterogeneity and typically follows an aggressive clinical course [1, 2]. Chronic active B-cell receptor (BCR) signaling has been implicated in lymphomagenesis [3]. BCR crosslinking promotes interaction between SRC family kinases (e.g., LYN) and CD79A/B and thereby induces activation of spleen tyrosine kinase (SYK). SYK is an integral BCR-signaling kinase that recruits Bruton tyrosine kinase (BTK) and adaptor molecules triggering divergent downstream events that lead to propagation of AKT, MAPK, and NFκB signaling and upregulation of the Bcl-2 family proteins [4]. Activation of the BCR-associated kinases SYK and BTK plays a pivotal role in regulation of survival, proliferation, and homing of malignant B cells.

In the past decade, small molecules targeting BTK (ibrutinib, acalabrutinib, zanubrutinib) received regulatory approvals for the treatment of MCL. Nevertheless, median duration of response is less than 2 years, and patients who develop therapeutic resistance have dismal outcomes [1, 5]. A retrospective study of 114 patients who developed ibrutinib resistance documented an overall survival of less than 3 months [5]. Acquired mutations in BTK (e.g., C481S) do not fully explain ibrutinib resistance and are only found in a subset of cases [6, 7]. A number of alternative mechanisms have been implicated in BTK inhibitor resistance in MCL. Constitutive NFκB activation due to recurrent mutations in *TRAF2* and *BIRC3* has been linked to primary ibrutinib refractoriness [8, 9], and therapeutic strategies which abrogate NFκB have shown promise in the pre-clinical setting [10, 11]. Interplay between the tumor microenvironment-driven phosphoinotiside-3-kinase/AKT/mTORC1 and integrin-β1/mTORC2 pathways also induces an ibrutinib-resistant phenotype [6, 12]. This premise justifies the exploration of novel therapeutic targets within and outside of the BCR-signaling cascade and design of rational drug combinations in MCL.

CG-806 (luxeptinib) is an investigational non-covalent kinase inhibitor that has entered early phase clinical trials for patients with relapsed or refractory hematological malignancies. CG-806 targets key BCR-associated kinases LYN, SYK, and BTK. Importantly, CG-806 potently inhibits both wildtype and C481S mutant BTK with IC_50_ 8 and 2.5 nM, respectively [13], and thus is expected to have activity in settings where resistance to BTK inhibitors is driven by these mutations. In a Phase I trial in patient with chronic lymphocytic leukemia (CLL) and NHL, treatment with CG-806 resulted in decreased phosphorylation of SYK and BTK in the circulating malignant cells within 8 h of administration [14]. Here we investigated the pre-clinical efficacy of the dual BTK/SYK inhibitor CG-806 in MCL.

## Materials and methods

### Cell lines, primary cells, and subjects

MCL cell lines Mino and JeKo-1 (both harboring *TP53* aberrations) and germinal center (GC) diffuse large B-cell lymphoma (DLBCL) cell line SU-DHL10 were obtained from American Type Culture Collective (ATCC) [15, 16]. GC-DLBCL cell lines U-2932, OCI-LY3 and activated B-cell type DLBCL cell lines OCI-LY19 and VAL were obtained from DSMZ (Braunschweig, Germany) [17]. Ibrutinib resistance was induced in Mino and JeKo-1 cell lines by exposure to increasing concentrations of ibrutinib (denoted MinoR and JeKo-1R) [11]. Cells were cultured in RPMI-1640 medium supplemented with 10% fetal bovine serum (FBS) except for Mino and Jeko-1 (20% FBS). Stromal cell lines L, HS-5 (ATCC) and L4.5 (DSMZ) were maintained in DMEM 1640 medium with 10% FBS. B-cell activating factor (BAFF)-expressing Chinese hamster ovary cells described previously were cultured in MEM-α supplemented with 10% FBS [18]. Mycoplasma testing was conducted every 3 months using the Mycoplasma PCR Detection Kit (Abcam).

Following approval by the Institutional Review Board and provision of written consent, peripheral blood was procured from 9 MCL patients (median age 65 years; 7 were male; 6 were previously untreated) followed at the Oregon Health & Science University (IRB# 4422). Peripheral blood mononuclear cells were isolated using the standard Ficoll-Hypaque technique (Amersham). ACK lysing buffer was used to lyse red blood cells (Life Technologies). Primary cells were cultured in RPMI-1640 medium supplemented with 20% FBS, 1% L-glutamine, 1% HEPES, and 1% nonessential amino acid solution. All culture media contained 100 U/mL penicillin and 100 μg/mL streptomycin.

Primary MCL cells were co-cultured with stroma as previously described [18]. Briefly, primary cells were plated at a 50:1 ratio on a 60–70% confluent stromal layer, incubated for 24 h and then treated with drugs. Prior to harvest, cells were washed from the stromal layer and transferred into a new well for 1 h to allow adhesion of residual stromal cells.

### In vivo MCL models

All animal studies were carried out in accordance with institutional guidelines (IACUC #IP00000158). Five-week-old nonobese diabetic/severe combined immunodeficiency/γCnull (NOD.Cg-Prkdc^scid^ Il2rg^tm1Wjl^/SzJ [NSG]) mice (The Jackson Laboratory) received 25 mg/kg busulfan via intraperitoneal injection. The following day, MCL cells (3 × 10^6^ in 200 µL PBS) were inoculated via tail vein injection. Two aggressive patient-derived xenograft (PDX) models were used: MCL96069 (*BIRC3*^L548fs^; further referred to as MCL-A) and MCL44685 (*ATM*^V1671fs^, *WHSC1*^E1009K^, *CREBBP*^Q2257H^; MCL-B) [19]. Circulating MCL cells are detected within 4 weeks (MCL-A) and 2 weeks of inoculation (MCL-B). If left untreated, mice succumb to the disease within 9–10 and 6 weeks, respectively.

Mice were bled weekly and blood was analyzed using flow cytometry to detect circulating MCL cells; red cells were lysed using ACK lysis buffer (Life Technologies), and the remaining cells were washed and stained with 0.5 µL LIVE/DEAD Fixable Aqua (Invitrogen), 20 µL CD19 (Becton Dickinson), 2 µL CD5, and 10 µL CD45 (Miltenyi Biotech) in 150 µL PBS then were analyzed by flow cytometry. MCL cells were classified as live lymphocytes (CD45^+^) that co-expressed CD5 and CD19 (aberrant concurrent expression of these markers is found on human MCL cells). Upon detection of circulating cells, mice were separated into groups and received treatment with CG-806 at either 30.8 or 308 mg/kg diluted in sodium lauryl sulfate (SLS) 8 mg/kg (*N* = 10 per group), ibrutinib 25 mg/kg diluted in 1% 2-hydroxypropyl-β-cyclodextrin (*N* = 5) or vehicle control (SLS 8 mg/kg; *N* = 10), daily via oral gavage.

Mice were followed for survival and were euthanized if weight loss exceeded 20%. At the time of sacrifice, spleen was homogenized via passage through a 40 µm cell strainer. Splenocytes were subjected to red blood cell lysis and analyzed for proliferation (Ki-67; Inside Stain Kit, Miltenyi Biotec) by flow cytometry. Total RNA was isolated using the E.Z.N.A. HP Total RNA Isolation Kit (Omega Biotek). Protein was harvested as described above. A sample size of ten animals in experimental arms yields >80% power for comparisons vs. control with a 5% significance level.

### Statistical analysis

All experiments were conducted at least in biological triplicates. Statistical significance was determined by Student’s *t*-test using GraphPad Prism software unless noted otherwise. *p < 0.05 and **p < 0.01 throughout the manuscript.

## Results

### Dual BTK/SYK inhibitor CG-806 downregulates BCR signaling, Mcl-1, and Bcl-xL in MCL cells

**Fig. 1:**
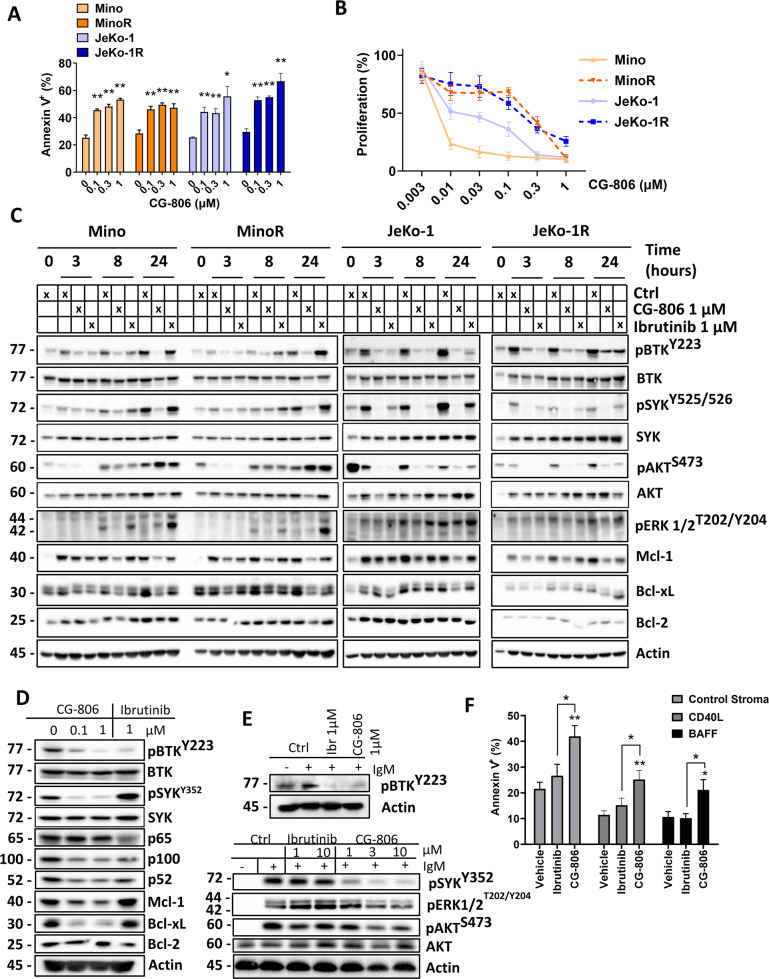
CG-806 induces apoptosis and attenuates B-cell receptor signaling in MCL. **A** Cells were treated with indicated doses of CG-806 for 24 h. Apoptosis was determined by Annexin-V-FITC staining. Data are shown as mean ± SEM. **B** Parental and ibrutinib-resistant MCL cell lines were treated with the indicated doses of CG-806 for 48 h. Cell proliferation was assessed using a colorimetric tetrazolium-based assay. Mean ± SEM is shown. **C** Cells were treated with drugs as indicated. Cell lysates were subjected to immunoblotting. **D** Mino cells were treated with the indicated concentrations of drugs for 24 h. **E** Primary MCL cells (from three individual patients) were treated with vehicle control, ibrutinib or CG-806 for 1 h, then were stimulated with 5 µg/mL IgM for 30 min. Whole-cell lysates were analyzed using immunoblotting. Representative blots are shown. **F** Primary MCL cells (*n* = 5 individual samples tested in technical duplicates) were co-cultured with stroma for 24 h. Cells were then treated with 1 µM CG-806 or ibrutinib for 48 h. Apoptosis of the CD19^+^ B-cell population was quantified using Annexin-V staining. *p < 0.05 and **p < 0.01 vs. untreated control.

Treatment with CG-806 restricted proliferation and induced apoptosis of parental and ibrutinib-resistant MCL cell lines (Fig. 1A, B) [11, 20]. Similarly, treatment with CG-806 diminished viability in a panel of four DLBCL cell lines (IC_50_~200 nM; Supplementary Fig. 1). MCL cell lines treated with CG-806 exhibited a decrease in phosphorylation of the proximal BCR-associated kinases SYK and BTK, which was sustained for 24 h and was more pronounced compared to ibrutinib (Fig. 1C, D and Supplementary Fig. 2). Both CG-806 and ibrutinib led to diminished AKT activation in Jeko-1 cells. Meanwhile, treatment with CG-806, but not ibrutinib, downregulated Erk phosphorylation (Fig. 1C).

In MCL, BCR signaling induces expression of the pro-survival Bcl-2 family member proteins in part via NFκB [3]. We observed downregulation of NFκB activity, in particular the non-canonical pathway, in Mino cells treated with kinase inhibitors (Fig. 1D and Supplementary Fig. 3A). Treatment with CG-806 downmodulated the pro-survival protein Bcl-xL in MCL cell lines, a bona fide NFκB signaling target (Fig. 1C, D) [21]. In addition, treatment with CG-806 but not ibrutinib led to reduced levels of Mcl-1. Disruption of BCR signaling with kinase inhibitors had no effect on Bcl-2 in this short-term in vitro assay.

Treatment with CG-806 inhibited BCR signaling in primary MCL cells (Fig. 1E and Supplementary Fig. 3B). While both ibrutinib and CG-806 reduced phospho-BTK, only CG-806 blocked SYK, AKT and ERK activation following IgM crosslinking.

Pro-survival signaling emanating from the stromal microenvironment contributes to drug resistance in MCL [22]. To partially mimic the lymph node microenvironment ex vivo, we employed CD40L- and BAFF (B-cell activation factor)-expressing stromal co-culture models that are known to upregulate NFκB signaling [18, 22, 23]. While CD40L-expressing stroma predominantly upregulates Bcl-xL, BAFF induces Mcl-1, rendering protection of primary MCL cells from spontaneous apoptosis [18]. BAFF- and CD40L-expressing stroma rendered protection from spontaneous apoptosis and induced ibrutinib resistance (*p* < 0.05). By contrast, treatment with CG-806 reversed this protective effect (Fig. 1F).

Thus, dual BTK/SYK inhibitor CG-806 blocked BCR signaling, downregulated Bcl-xL and Mcl-1, and thwarted stromal support in MCL cells.

### CG-806 induces mitochondrial damage and metabolic reprogramming

**Fig. 2:**
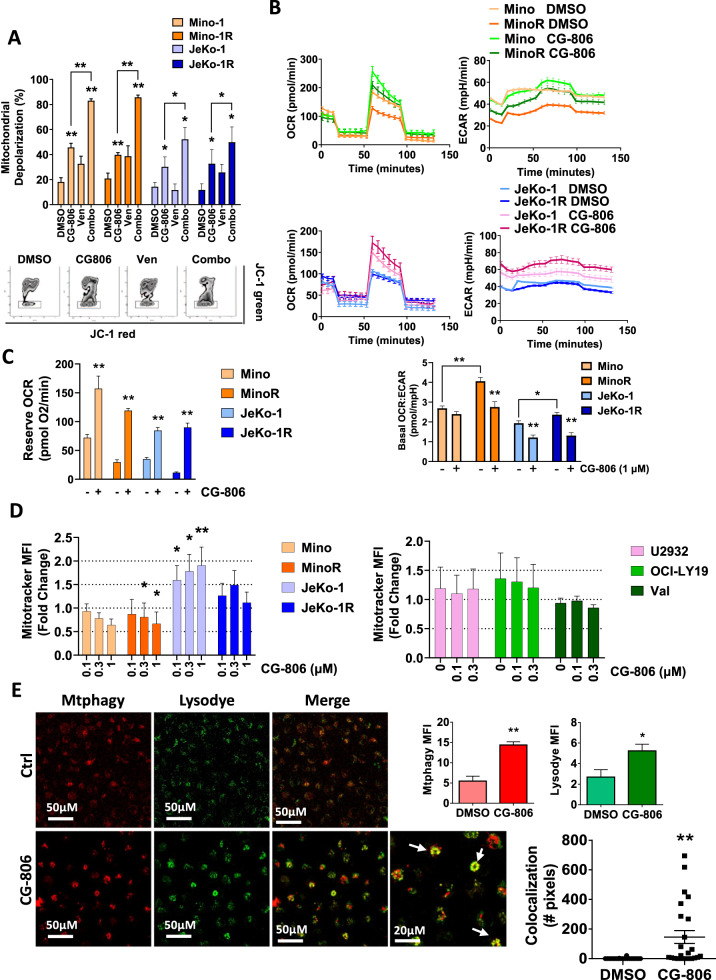
CG-806 induces mitochondrial damage and metabolic reprogramming. **A** Cells were treated with CG-806 (1 µM), venetoclax (Ven, 0.1 µM), or combination for 24 h and were analyzed for mitochondrial depolarization with JC-1 dye using flow cytometry. Data are presented as mean ± SEM. A representative dot plot image is also shown (depolarized mitochondria are boxed). **B**, **C** MCL cells were treated with 1 µM CG-806 or DMSO control for 24 h and subjected to Seahorse analysis measuring oxygen consumption rate (OCR) and extracellular acidification rate (ECAR). **D** Cell lines were treated with drug for 24 h as indicated and mitochondrial mass was assessed with Mitobright Green using flow cytometry. Data are presented as mean ± SEM. **E** Mino cells were treated with 1 µM CG-806 vs. control for 24 h and stained with Mtphagy dye (red) and Lysodye (green). Live cells were imaged with confocal microscopy. A representative image is shown. White arrows point to the mitophagy puncta. MFI was quantified, and colocalization was measured and calculated using Zen software. Data are presented as mean ± SEM and pixel count. *p < 0.05 and **p < 0.01 vs. control.

Bcl-2 family proteins control mitochondrial outer membrane permeabilization (MOMP) and thereby determine cell fate [24]. Since dual BTK/SYK inhibition altered the expression of Mcl-1 and Bcl-xL, we investigated the effects of CG-806 on mitochondrial dynamics. Treatment with CG-806 induced mitochondrial depolarization in MCL cell lines (Fig. 2A). The CG-806 effect was more pronounced compared with venetoclax, a selective Bcl-2 inhibitor, and was most prominent with a combination of the two drugs. We then examined oxygen consumption rate (OCR) and extracellular acidification rate (ECAR), the indirect measures of oxidative phosphorylation (OxPhos) and glycolysis, respectively. Measurements were taken at baseline and over time in response to chemical perturbation of oxidative metabolism. Ibrutinib-resistant MCL cell lines demonstrated an increase in basal OCR:ECAR ratio compared with parental cells, indicating increased reliance on OxPhos (Fig. 2B). By contrast, treatment with CG-806 led to heightened basal ECAR, while basal OCR remained unaffected, indicating a shift toward glycolytic metabolism (Fig. 2B). Consistent with this result, treatment with CG-806 had no significant effect on the generation of reactive oxygen species, a metabolic byproduct of OxPhos (Supplementary Fig. 4A) [25].

Reserve respiratory capacity, calculated as the difference between basal and uncoupled maximal OCR, impacts cell survival under conditions of metabolic stress [26]. CG-806 treatment led to a dramatic increase (2.5–22 fold) in reserve respiratory capacity in all tested MCL cell lines (Fig. 2C). An increase in mitochondrial mass may augment respiratory capacity. However, treatment with CG-806 induced variable but overall minimal changes in mitochondrial mass across a panel of MCL and DLBCL cell lines (Fig. 2D).

Finally, we probed for mitophagy, a conserved process whereby damaged mitochondria are sequestered by autophagosomes then fused to lysosomes for degradation [27]. Treatment with CG-806 induced mitophagy, as evidenced by increased staining for damaged mitochondria and colocalization of mitochondria with lysosomes (Fig. 2E and Supplementary Fig. 4B).

Taken together, these data suggest that kinase inhibition with CG-806 induced mitochondrial damage and metabolic reprogramming toward glycolysis with a concurrent increase in reserve respiratory capacity.

### CG-806 blocks BCR signaling in vivo

**Fig. 3:**
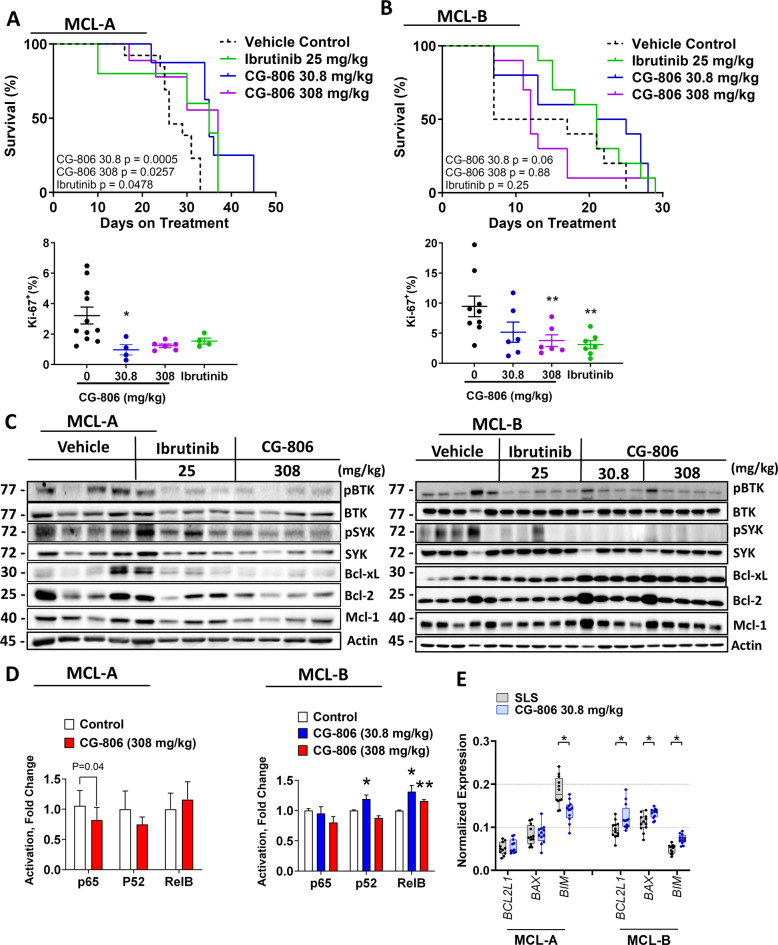
CG-806 restricts MCL development and BCR signaling in vivo. **A**–**C** Mice were inoculated with MCL cells as described in the methods. Two distinct MCL PDX models were used. Once circulating CD5/CD19+ MCL cells were detected in the peripheral blood, mice began daily treatment with CG-806, ibrutinib, or vehicle control via oral gavage in the indicated doses. Kaplan–Meier survival curves are shown, significance determined by log-rank test. At the end of the experiment, mice were euthanized 2 h after the final drug dose, and spleen-resident tumor cells were analyzed for proliferation and were subjected to immunoblotting. **D** NF-κB activation in splenocytes from MCL-A (*N* = 8) and MCL-B (*N* = 5) mice was quantified using the TransAm NF-κB Activation Assay. **E** mRNA expression of the indicated gene transcripts was quantified using RT-PCR and normalized to GAPDH. Data are shown as a box and whisker plot with dots representing expression values from four mice. *p < 0.05 and **p < 0.01 vs. vehicle control.

We next investigated whether CG-806 blocks activation of BCR-associated kinases in MCL in vivo. Two distinct PDX models, both originating from patients with multi-drug resistant MCL, were used. In model MCL-A, circulating tumor cells were detected 4–5 weeks after inoculation and treatment with CG-806 began at that time. Treatment led to slightly prolonged survival of the xenografted mice and suppressed proliferation of spleen-resident malignant cells (Fig. 3A). By contrast, in the MCL-B model, circulating tumor cells were detected within 2 weeks of inoculation. These cells exhibited a higher baseline proliferation index than MCL-A (Fig. 3B). While treatment with kinase inhibitors suppressed the proliferation of malignant splenocytes, this did not translate into improved survival in the MCL-B model (Fig. 3B). Treatment with CG-806 did not result in weight loss or other noticeable toxicities.

Consistent with our in vitro findings, in vivo treatment with CG-806 suppressed BTK and SYK activity in the xenografted splenocytes in both models (Fig. 3C and Supplementary Fig. 5). Treatment with CG-806 suppressed the expression of Mcl-1 and Bcl-2 in the drug-sensitive MCL-A model. By contrast, this effect was not seen in the resistant model (MCL-B) treated with either kinase inhibitor. In fact, Bcl-xL expression was upregulated in the CG-806-treatmed group (Fig. 3C). BCR signaling is the key driver of NFκB which in turn induces Bcl-2 proteins. Treatment with CG-806 led to a reduction of canonical NFκB signaling and a trend toward lower non-canonical signaling (p52) in the MCL-A model (Fig. 3D). By contrast, NFκB was not inhibited and in fact was slightly upregulated in the resistant MCL-B model. MCL-A model mice treated with CG-806 had reduced or unchanged mRNA expression of three known NFκB target genes, whereas MCL-B mice showed increased expression (Fig. 3E). Thus, BCR-independent activation of the pro-survival pathways, including NFκB, may be responsible for upregulation of Bcl-xL, contributing to resistance to dual BTK/SYK inhibition.

**Fig. 4:**
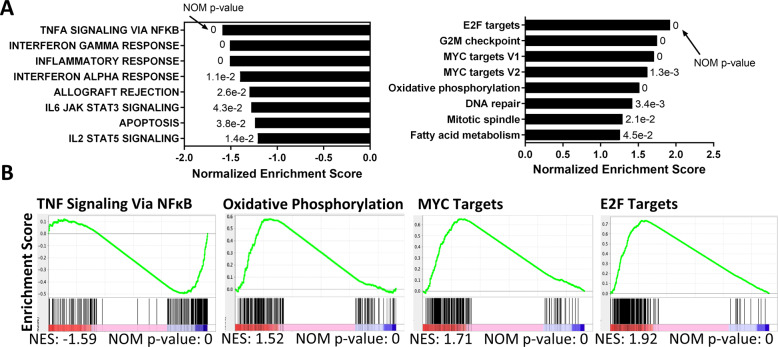
Transcriptional effects of dual SYK/BTK inhibition in vivo. Spleen-resident tumor cells of the MCL-B PDX model were harvested 2 h after the final treatment with CG-806 at 308 mg/kg or control as described in the methods. Gene set enrichment of RNA was calculated using gsea2–2.24. *N* = 3. **A** Representation of the top 8 pathways that were differentially regulated in the treatment vs. control samples. **B** Enrichment plots of select gene sets. Differentially regulated genes were defined by log_2_fc ≥ 0.58/≤−0.58 and *P*adj <0.05.

We next sought to gain additional insight into pathways deregulated by dual BTK/SYK inhibition in the resistant MCL PDX model. RNA-seq analysis was performed on splenocytes harvested from MCL-B mice treated with CG-806 (308 mg/kg) or vehicle control (*n* = 3 each). Using a cut-off of log_2_fc ≥ 0.58/≤−0.58, and *p*-adjusted <0.05, unsupervised hierarchical clustering revealed 278 genes that were differentially expressed in the CG-806-treated cohort vs. vehicle control, of which 161 were downregulated while the 117 were upregulated (Supplementary Fig. 6). Gene set enrichment analysis of significantly differential genes pointed toward transcriptional downregulation of JAK/STAT and interferon response genes (Fig. 4A). While “TNF signaling via NFκB” was significantly downregulated, a subset of NFκB gene targets were upregulated (Fig. 4B). Featured amongst the significantly upregulated gene sets were metabolic pathways (OxPhos, fatty acid metabolism), MYC targets, and cell cycle-related pathways.

Thus, while treatment with CG-806 blocks BCR signaling in vivo, resistance mechanisms may involve NFκB signaling and upregulation of Bcl-xL and other oncogenic pathways.

### Genetic and functional screening assays implicate the Bcl-2 network in response to BTK/SYK inhibition

**Fig. 5:**
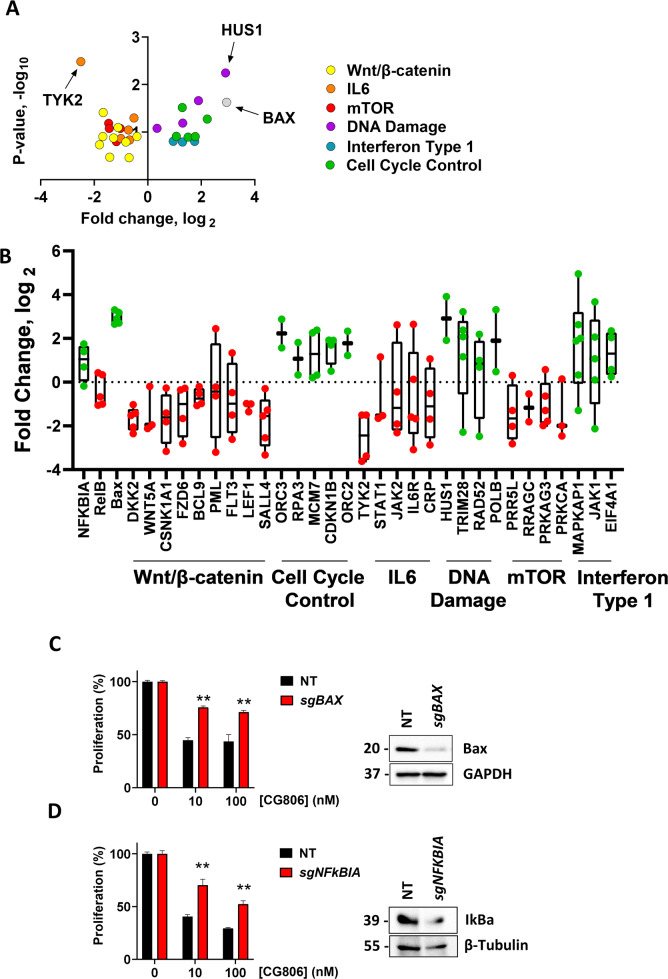
Genome-wide CRISPR-Cas9 library screen implicates BAX and NFKBIA loss in resistance to CG-806. Genome-wide loss of function CRISPR library screening was carried out in OCI-LY3 cells as described in the methods. Data were analyzed using a MaGeCK pipeline analysis. **A** Volcano plot of mid fold change vs. mid *p* value of all sgRNA per gene in select genes grouped by the association in pathways identified using webgestalt software. Highly differential and significant genes are identified with black arrows. **B** Box and whisker plot spanning min/max fold change of CG-806 vs control with individual sgRNA per gene depicted as points. Genes for which a majority of sgRNA demonstrate positive fold change are shown in green, negative in red. **C**, **D** BAX and NFKBIA knockout was established in Jeko-1 cells using RNP electroporation as described in the methods. Whole-cell lysates were subjected to immunoblotting. Cells were treated with CG-806 or vehicle control at the indicated concentrations for 48 h. Cell proliferation was assessed using a colorimetric tetrazolium-based assay. Mean±SE is shown. *p < 0.05 and **p < 0.01 vs. NT control.

To further elucidate which genes and pathways mediate sensitivity and resistance to CG-806, we conducted a genome-wide loss of function CRISPR-Cas9 library screen. Cas9-expressing OCI-LY3 cells were transduced with a lentivirus encoding a CRISPR library comprised of single-guide RNAs (sgRNA) targeting 18,010 genes with an average of 5 unique sgRNA per gene. Stably transduced cells were treated with DMSO or 1 µM CG-806 for 7 days. DNA was extracted and barcodes corresponding to sgRNA were PCR amplified and sequenced. sgRNA fold change was calculated in CG-806 vs. control. sgRNA targeting genes in the DNA damage, interferon type I, and cell cycle control pathways were enriched in cells treated with CG-806, suggesting that inactivation of these pathways promotes CG-806 resistance (Fig. 5A, B). The pro-apoptotic pore-forming Bcl-2 family member *BAX* arose as one of the most significantly enriched sgRNA. To validate this result, Jeko-1 cells were electroporated with Cas9 and *BAX*-targeting sgRNA (Fig. 5C and Supplementary Fig. 7). Loss of *BAX* conferred resistance to CG-806, supporting our earlier data implicating the importance of anti-apoptotic Bcl-2 family members and MOMP in CG-806-induced apoptosis.

Examining the average log fold change for individual sgRNAs revealed enrichment of *NFKBIA* sgRNA in the CG-806 treated cell population (Fig. 5B). *NFKBIA* encodes the IκBα protein that inhibits nuclear translocation of NFκB effectors, and thus serves as a negative pathway regulator. Jeko-1 cells electroporated with pooled sgRNAs targeting the *NFKBIA* gene demonstrated decreased sensitivity to CG-806, implicating NFκB signaling in resistance to CG-806 (Fig. 5D and Supplementary Fig. 7) [28]. Finally, sgRNA targeting genes in the Wnt/β-catenin and mTOR signaling pathways demonstrated negative fold change in the genome-wide loss of function screen, suggesting that they may contribute to resistance to CG-806 (Fig. 5A, B).

**Fig. 6:**
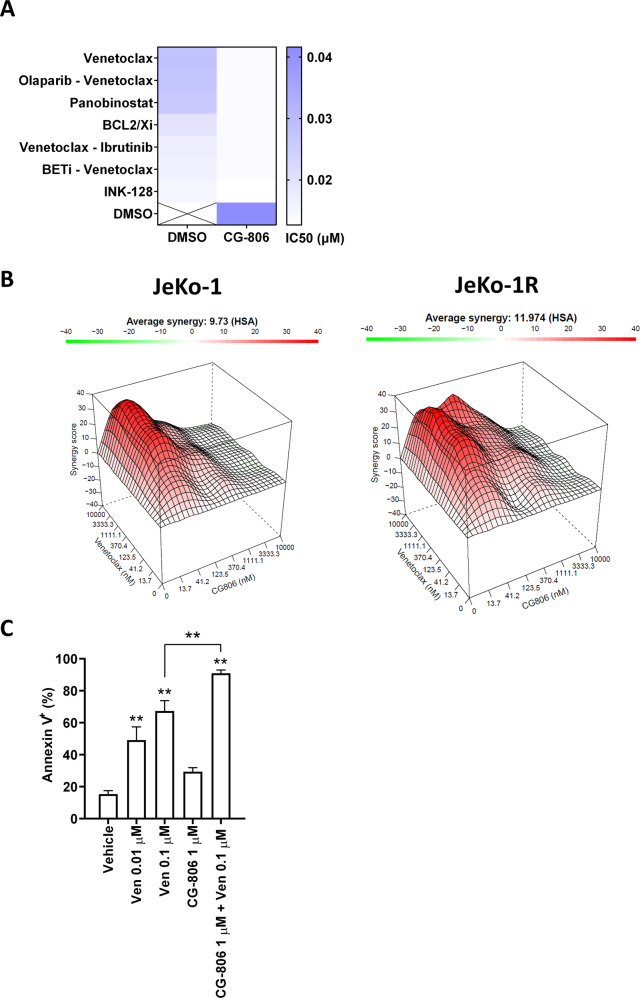
Concurrent Bcl-2 inhibition sensitizes NHL cells to dual SYK/BTK inhibitor G-806. **A** U-2932 cells were subjected to functional screening assay as described in the methods. IC50 values were calculated (represented in the color gradient). Drugs that exhibit synergy with CG-806 are shown. **B** Visualization of calculated synergy map based on the dose-response matrix. JeKo-1 and JeKo-1R cells were treated with CG-806, venetoclax, or a combination of the two in an 7 × 7 matrix as indicated for 48 h. Cell proliferation was measured using a colorimetric tetrazolium-based assay; % viable cells were normalized to DMSO-treated control. **C** Primary MCL cells (*N* = 4 individual samples analyzed in technical duplicates) were co-cultured with HS-5 stroma for 24 h. Cells were then treated with drugs for 24 h. Apoptosis of the CD19^+^ B-cell population was quantified using Annexin-V staining. *p < 0.05 and **p < 0.01 vs. vehicle control.

Next, we employed a high-throughput drug-screening assay comprised of a panel of 189 small molecule inhibitors known to target oncogenic pathways in order to identify agents that demonstrate a cooperative effect with CG-806 (see Methods). A proprietary Bcl-2/xL inhibitor, histone deacetylase inhibitor panobinostat, and mTOR pathway inhibitor INK-128 served as sensitizers to BTK/SYK inhibition (Fig. 6A). Consistent with the screening assay results, CG-806 cooperated with Bcl-2 inhibitor venetoclax in a 7 × 7 matrix MTS assay in JeKo-1, JeKo-1R, and U-2932 cells (Fig. 6B and Supplementary Fig. 8A). Interestingly, ibrutinib-resistant MCL cells demonstrated relative resistance to venetoclax compared to their parental counterparts (Supplementary Fig. 8B). Combined treatment with venetoclax enhanced CG-806-mediated loss of mitochondrial membrane potential in MCL cells (Fig. 2A). Lastly, we studied synergy between CG-806 and venetoclax in primary MCL cells. Combined exposure to venetoclax and CG-806 showed a robust killing effect, with 91% apoptosis in stromal conditions (Fig. 6C).

Together, these results show that the Bcl-2 network governs susceptibility to CG-806 and implicates it as a target for combination therapy with CG-806.

## Discussion

B-cell malignancies are governed by a complex milieu of regulatory networks that regulate the equilibrium of cell proliferation and apoptosis. SYK, a kinase indispensable in BCR signaling, additionally transmits the pro-survival signals emanating from the lymph node microenvironment (e.g., BAFF and CD40L), and is, therefore, an attractive target in NHL [18]. Constitutive activation of LYN and SYK has been shown to promote BTK-independent activation of the BCR cascade in ibrutinib-resistant lymphoid tumors [29, 30]. An ever-growing body of evidence implicates upregulation of the pro-survival proteins Mcl-1 and Bcl-xL in resistance to Bcl-2 inhibitor venetoclax [31–33], and SYK inhibition emerged as a potential approach to circumvent resistance to ibrutinib and venetoclax in pre-clinical models [29, 30, 32]. Fostamatinib, a non-selective SYK inhibitor approved in treatment of immune thrombocytopenia, has demonstrated clinical efficacy in NHL [34, 35]. We reported that selective investigational SYK inhibitor entospletinib was safe and efficacious in patients with CLL when combined with obinutuzumab, an anti-CD20 antibody, or tirabrutinib, a BTK inhibitor [36, 37]. A combination of entospletinib and tirabrutinib was associated with an overall response rate of 64% in a small study of 11 patients with heavily pre-treated MCL [38]. Therefore, continued clinical exploration of kinase inhibitors that target SYK and BTK is highly relevant.

Here, we show that dual BTK/SYK inhibitor CG-806 thwarted proliferation and induced apoptosis in parental and ibrutinib-resistant MCL cell lines. Treatment with CG-806 attenuated BTK signaling in MCL cell lines and primary cells. In contrast to ibrutinib, CG-806 also blocked activation of SYK and inhibited ERK phosphorylation. These events, observed with CG-806 treatment but not ibrutinib, likely accounted for loss of Mcl-1, since we and others have shown that SYK and Erk are necessary to sustain Mcl-1 transcription and protein stability, respectively [18, 32]. Both kinase inhibitors repressed Bcl-xL, an NFκB target, although CG-806 had a more pronounced effect, possibly due to downregulation of non-canonical NFκB signaling. The suppression of anti-apoptotic Bcl-2 family members likely underlies the superior ability of CG-806 to abrogate survival of MCL cells in the lymph node-mimicking conditions, i.e., on BAFF and CD40L-expresing stroma, where induction of Bcl-xL and Mcl-1 is known to rescue neoplastic B cells from spontaneous and drug-induced apoptosis [18, 23]. Finally, dually targeting BTK/SYK with CG-806 demonstrated efficacy in a PDX MCL model, where efficacy was accompanied by downmodulation of Bcl-2 family proteins and NFκB.

Metabolic reprogramming, often toward heightened aerobic glycolysis (the Warburg effect), is a hallmark of cancer. While the impact of BCR-associated kinase inhibitors on cellular bioenergetics has not been well studied, emerging evidence suggests that metabolic state may impact therapeutic outcomes, and that cellular bioenergetic responses and BTK activity are interdependent. Ibrutinib was shown to induce a metabolic stress response in primary neoplastic lymphocytes, and in vitro drug sensitivity was modulated following perturbations in cellular bioenergetics [39, 40]. In another study, CLL cells obtained from patients treated with ibrutinib exhibited a decreased OCR, on par with that of normal lymphocytes [41]. Here we demonstrate that BTK/SYK inhibition with CG-806 induces mitochondrial damage and leads to metabolic reprogramming in neoplastic B cells. Consistent with work by others, we observed an increased baseline OCR:ECAR ratio in ibrutinib-resistant MCL cell lines, suggesting increased reliance on oxidative metabolism [42, 43]. By contrast, cells treated with CG-806 exhibited a glycolytic shift, accompanied by mitochondrial depolarization and induction of mitophagy, a conserved process that eliminates dysfunctional mitochondria.

Despite the mitochondrial damage, CG-806 treatment dramatically increased the respiratory reserve capacity, a phenomenon that could result from either increased mitochondrial mass, enhanced substrate availability or electron transport chain integrity [44]. CG-806-treated cells did not exhibit a consistent change in mitochondrial mass, and electron transport chain integrity was likely compromised due to MOMP. Hence, it is probable that an observed increase in respiratory capacity is a result of increased substrate availability. Interestingly, ibrutinib was previously shown to upregulate glucose and glutamine uptake in primary CLL cells [43]. Respiratory reserve capacity buffers cells from conditions of metabolic stress, and therefore may protect malignant cells from the consequences of mitochondrial damage.

Transcriptomic analysis of MCL cells from the resistant PDX model identified OxPhos as one of the highly upregulated gene sets. This is consistent with published evidence implicating OxPhos in resistance to targeted agents (including ibrutinib and the Bcl-2 inhibitor venetoclax) in lymphoid malignancies [42, 45]. Thus, OxPhos is a relevant therapeutic target in MCL alone or in addition to BCR-associated kinases. We also found that MYC transcriptional program was highly upregulated in the resistant MCL cells. MYC plays an intimate role in regulation of metabolism, and has also been linked to ibrutinib resistance in MCL, not in the least via induction of OxPhos [12, 42, 46]. MYC promotes glutamine anaplerosis into the TCA cycle and thereby drives mTOR signaling, which then causes additional downstream metabolic changes [47]. Further study is required to determine whether MYC orchestrates metabolic reprogramming to mediate resistance to dual BTK/SYK inhibition in MCL in coordination with other oncogenic pathways.

In a CRISPR-cas9 screen cell cycle control and DNA damage response pathways were found to confer sensitivity to CG-806, both of which showed transcriptomic upregulation in an MCL PDX model treated with CG-806. Conversely, mTOR and Wnt/β-catenin signaling arose as influential oncogenic factors. *BAX*, a direct activator of apoptosis, and *NFKBI*, a negative NFκB signaling regulator, were identified as genes that modulate sensitivity to BTK/SYK inhibition. This corresponded to our in vivo findings demonstrating that a subset of NFκB target genes including Bcl-xL were upregulated in the resistant PDX model and may be due to increased DNA-binding activity of the non-canonical NFκB family member RelB. Genetic knockout of *BAX* and *NFKBIA* reduced sensitivity of MCL cells to CG-806, confirming the role of the NFκB pathway and Bcl-2 family network in resistance to dual BTK/SYK inhibition. Furthermore, Bcl-2 inhibitors demonstrated synergy with CG-806 in a functional drug screen, including in ibrutinib-resistant MCL cells. Clinical development of therapeutic strategies will be guided by efficacy and toxicity. The combination of a BTK/SYK inhibitor and novel BH3-mimetics may be of particular interest given highly promising results of concurrent BTK and Bcl-2 targeting in lymphoid malignancies [48].

Taken together, our results demonstrate that dual BTK/SYK inhibition with CG-806 is a promising new therapy in MCL and NHL.

## Supplementary information

Supplementary Material

Supplementary Methods

Authorship Change

Reproducibility checklist

## Data Availability

Full results of the RNA-Seq analysis are available at https://www.ncbi.nlm.nih.gov/geo/query/acc.cgi?acc=GSE173353.
