# Supplementary material for: Dual BTK/SYK inhibition with CG-806 (luxeptinib) disrupts B-cell receptor and Bcl-2 signaling networks in mantle cell lymphoma
Source: Cell Death Dis. 2022 Mar 16;13(3):246. doi: 10.1038/s41419-022-04684-1 (PMC8927405; doi:10.1038/s41419-022-04684-1)
Supplement: Supplementary file 1 — Supplementary Material [file 41419_2022_4684_MOESM1_ESM.pptx]

## Slide 1
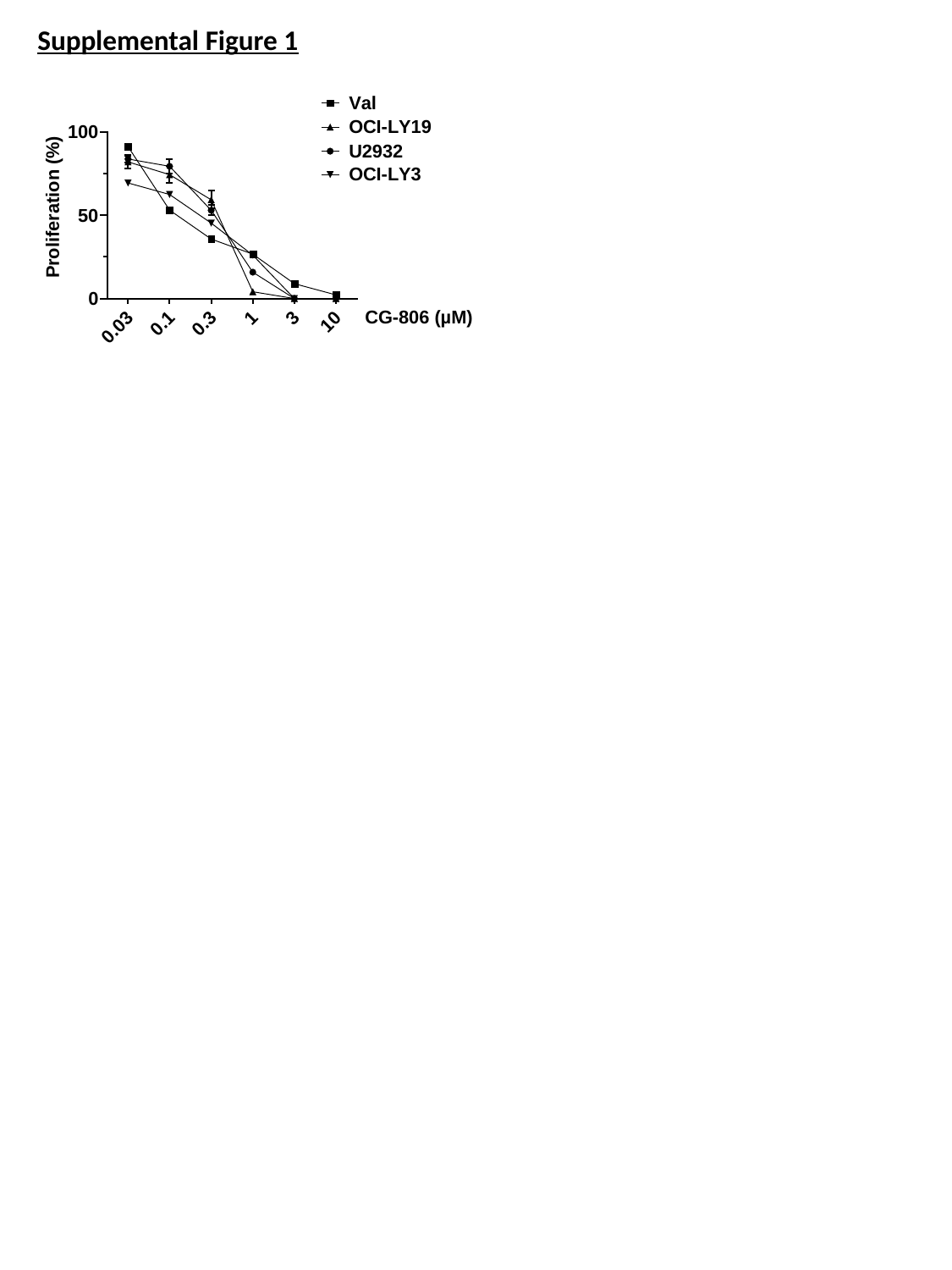

Supplemental Figure 1

## Slide 2
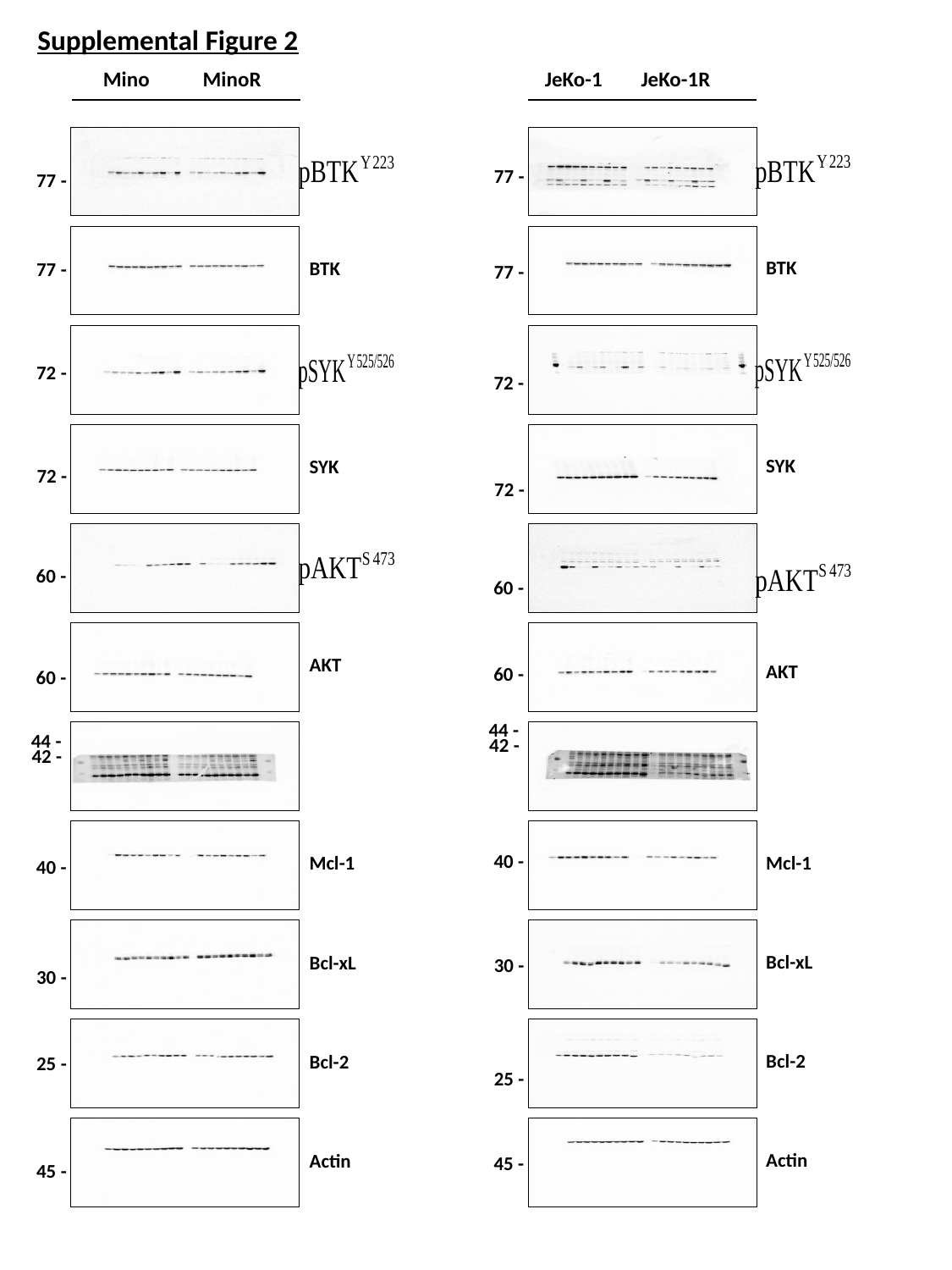

Supplemental Figure 2
JeKo-1 JeKo-1R
Mino MinoR
77 -
77 -
BTK
BTK
77 -
77 -
72 -
72 -
SYK
SYK
72 -
72 -
60 -
60 -
AKT
AKT
60 -
60 -
44 -
44 -
42 -
42 -
40 -
Mcl-1
Mcl-1
40 -
Bcl-xL
Bcl-xL
30 -
30 -
Bcl-2
Bcl-2
25 -
25 -
Actin
Actin
45 -
45 -

## Slide 3
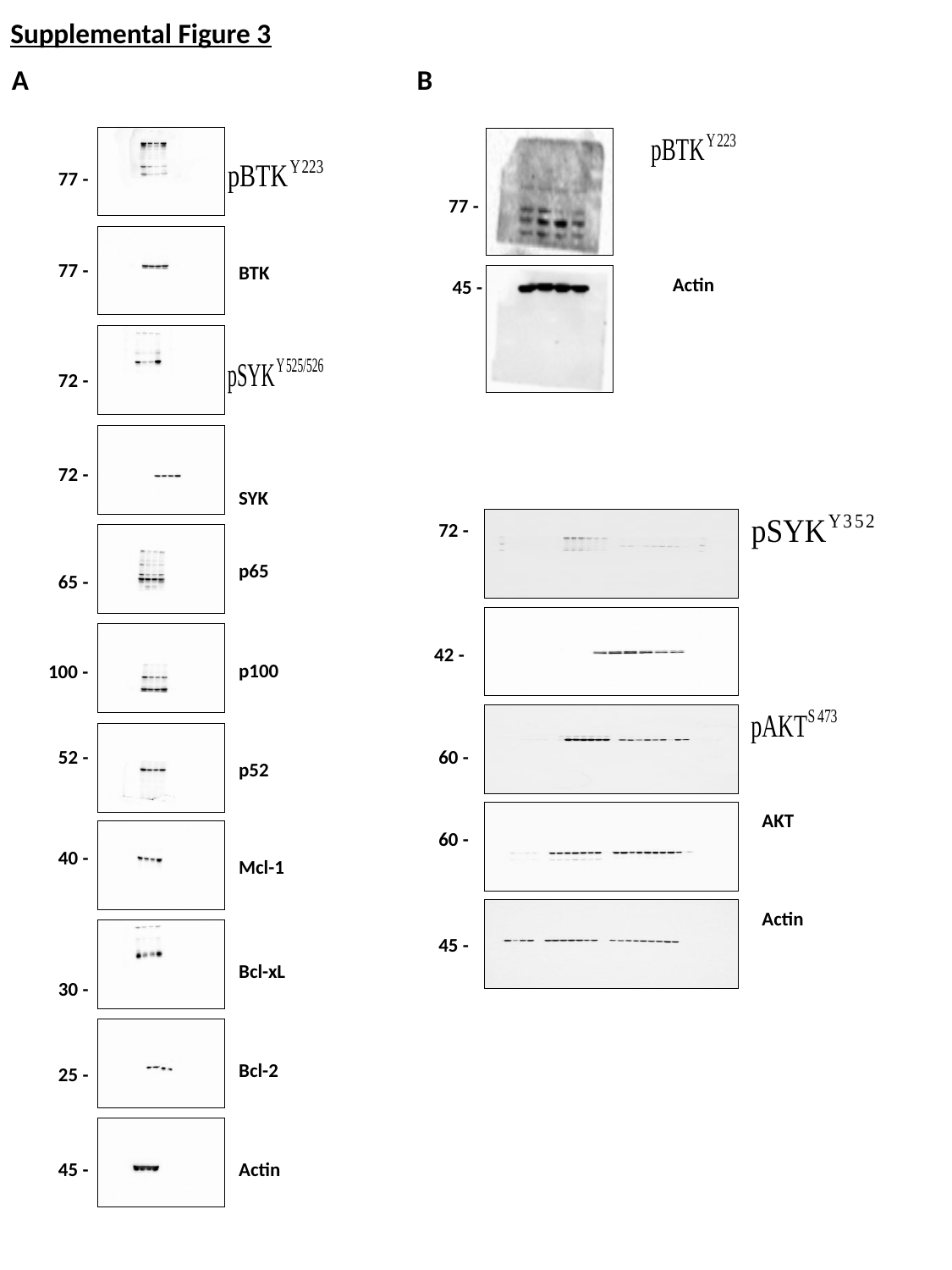

Supplemental Figure 3
A
B
77 -
77 -
77 -
BTK
Actin
45 -
72 -
72 -
SYK
72 -
p65
65 -
42 -
p100
100 -
60 -
52 -
p52
AKT
60 -
40 -
Mcl-1
Actin
45 -
Bcl-xL
30 -
Bcl-2
25 -
Actin
45 -

## Slide 4
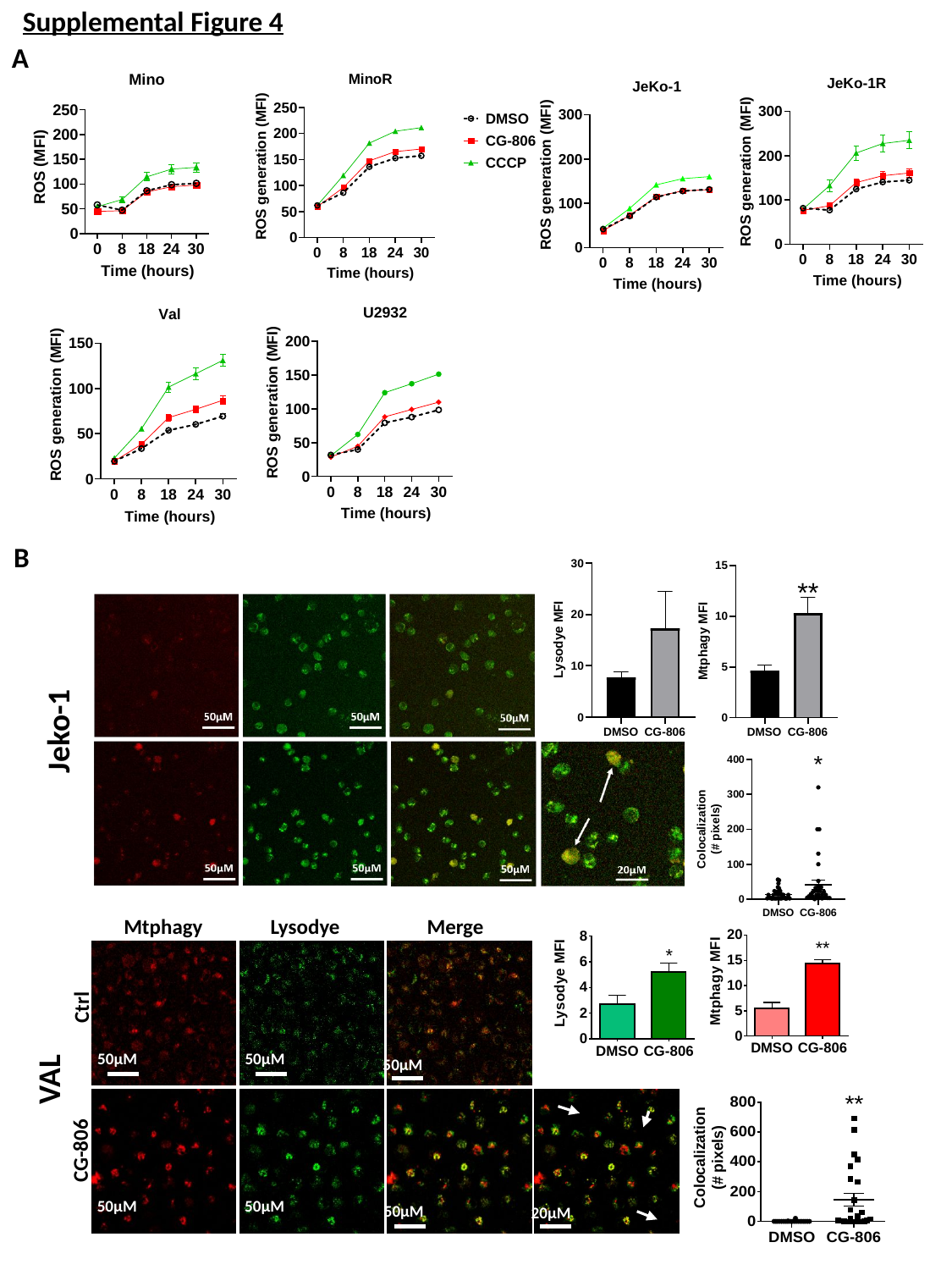

Supplemental Figure 4
A
B
Jeko-1
Mtphagy Lysodye Merge
 Ctrl
50µM
 CG-806
50µM
20µM
50µM
50µM
VAL
50µM
50µM

## Slide 5
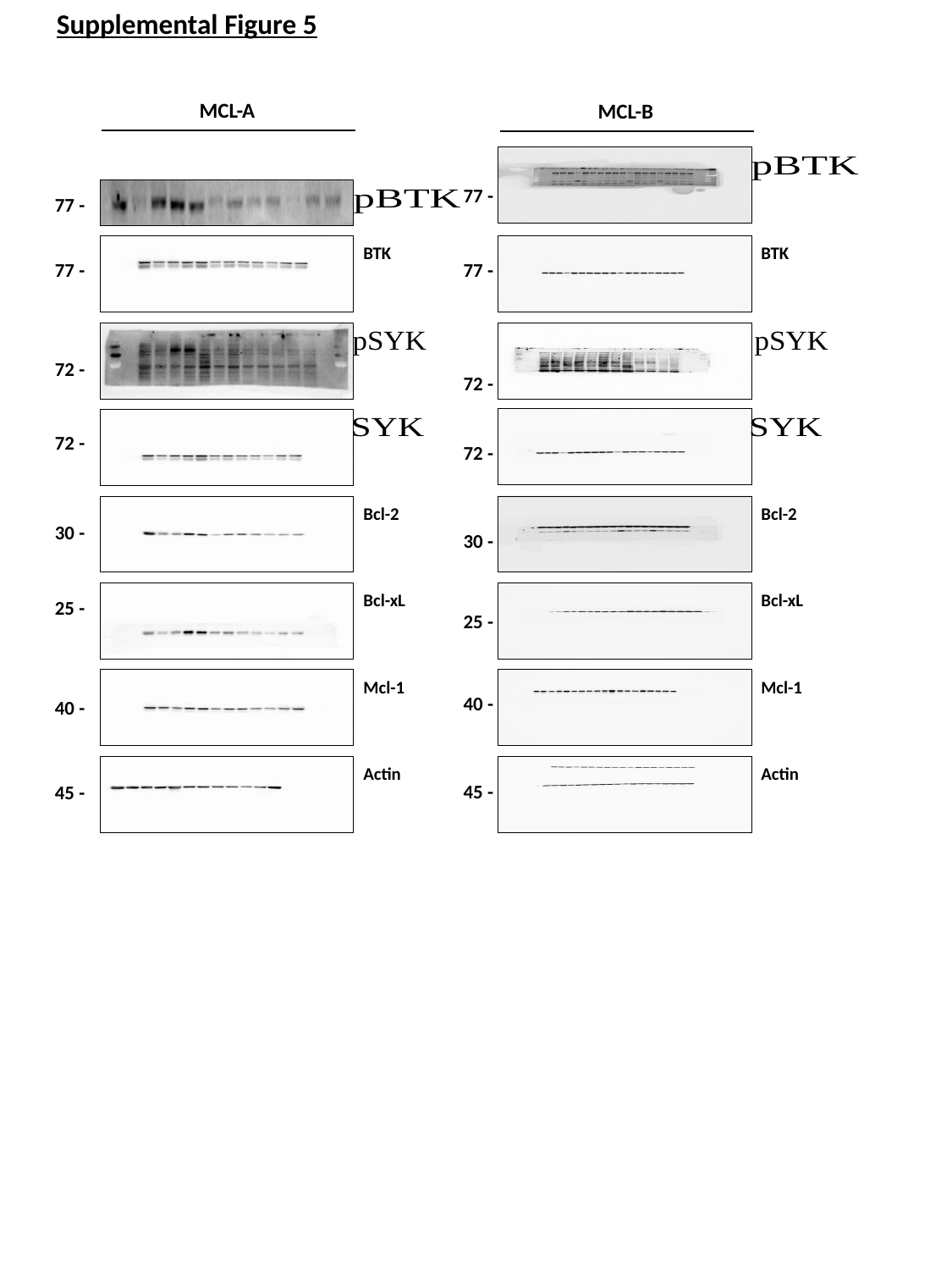

Supplemental Figure 5
MCL-A
MCL-B
77 -
77 -
BTK
BTK
77 -
77 -
72 -
72 -
72 -
72 -
Bcl-2
Bcl-2
30 -
30 -
Bcl-xL
Bcl-xL
25 -
25 -
Mcl-1
Mcl-1
40 -
40 -
Actin
Actin
45 -
45 -

## Slide 6
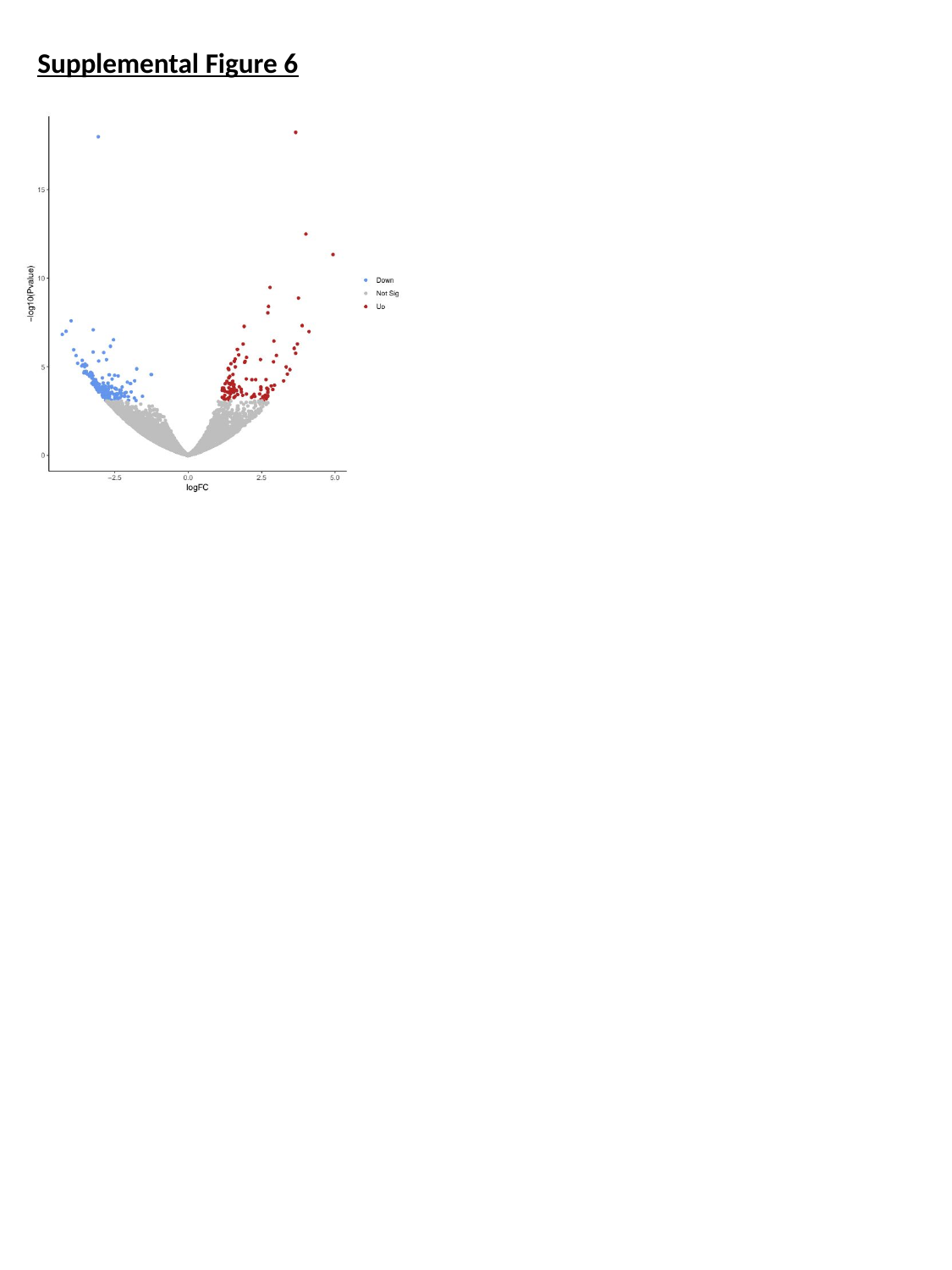

Supplemental Figure 6

## Slide 7
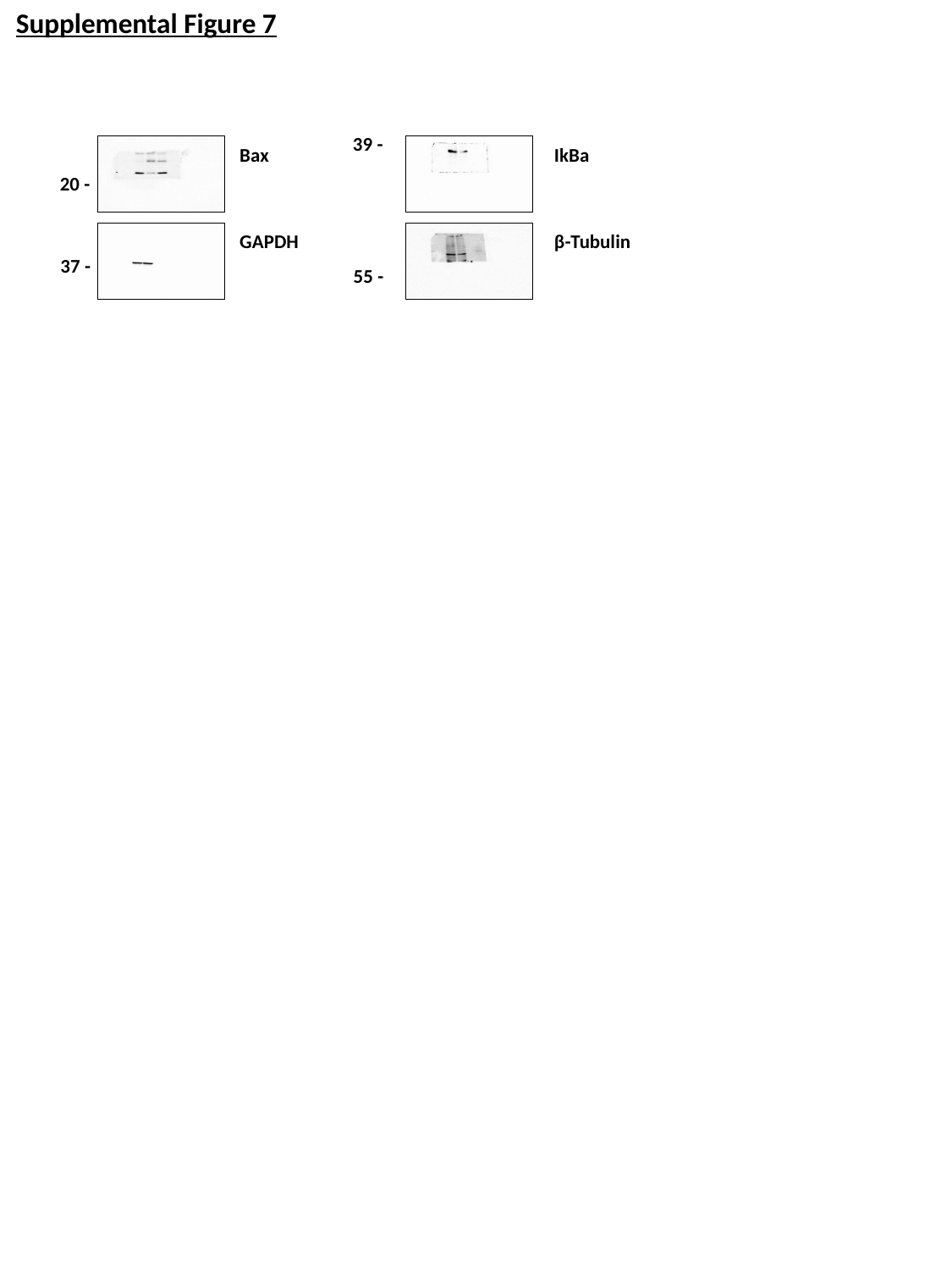

Supplemental Figure 7
39 -
Bax
IkBa
20 -
GAPDH
β-Tubulin
37 -
55 -

## Slide 8
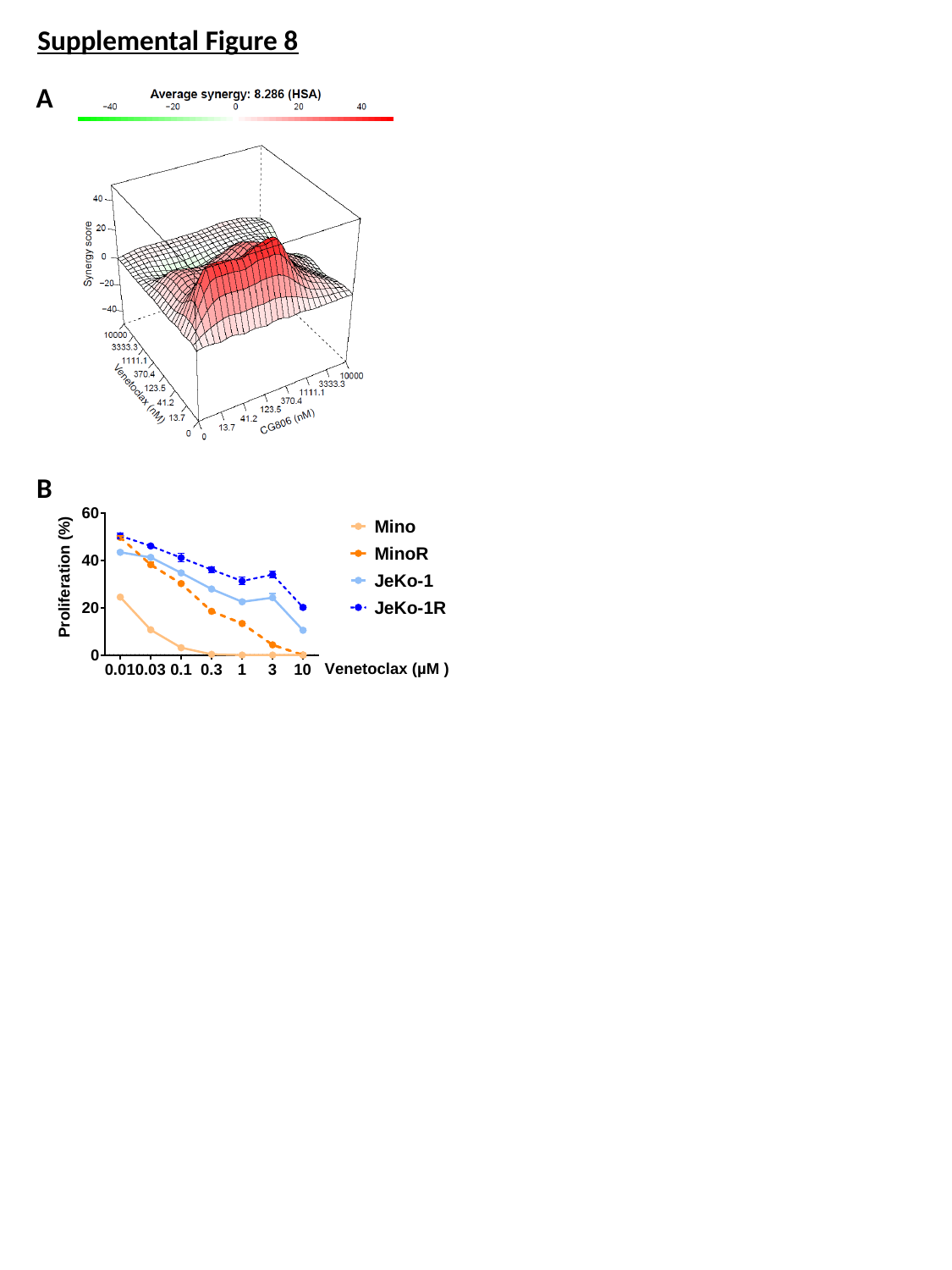

Supplemental Figure 8
A
B
