# Supplementary material for: Dual BTK/SYK inhibition with CG-806 (luxeptinib) disrupts B-cell receptor and Bcl-2 signaling networks in mantle cell lymphoma
Source: Cell Death Dis. 2022 Mar 16;13(3):246. doi: 10.1038/s41419-022-04684-1 (PMC8927405; doi:10.1038/s41419-022-04684-1)
Supplement: Supplementary file 2 — Supplementary Methods [file 41419_2022_4684_MOESM2_ESM.docx]

**Supplemental Methods**

*Viability testing and drugs*

Proliferation was evaluated using a tetrazolium-based colorimetric assay. Cells were seeded in a 96 or 384 well plate at 5000 or 1250 cells per well, respectively. Cells were treated with CG-806 and/or venetoclax at a dose range of 0-1 µM. This range was chosen because all cell lines tested demonstrated a complete loss of proliferation at 1 µM of CG-806 or venetoclax alone. Following incubation with drugs at 37°C in 5% CO_2_, methanethiosulfonate (MTS) reagent (CellTiter96 AQ_ueous_ One, Promega) was added and cells were incubated for an additional 4 hours before measurement of optical density at 490 nm.

Cell apoptosis was measured in duplicates as previously described using the ApoScreen Annexin V Apoptosis Kit.^1^ Briefly, cells were resuspended in 100 µL of Annexin V binding buffer containing 0.5 µL of Annexin V and 0.5 µL of 7-aminoactinomycin D (7-AAD). 1 µL of CD19 mAbs was added when primary lymphocytes were studied (Southern Biotech). Fluorescence was quantified using flow cytometry on LSRFortessa™ or FACSAria™ (BD Biosciences). Data was analyzed using FlowJo software (Tree Star).

CG-806 was provided by Aptose Biosciences; venetoclax was obtained from MedChemExpress.

*Immunoblotting*

Cells were lysed in Tris-HCL pH 7.4 (20 mM), NaCl (150 mM), EDTA (1 mM), EGTA (1 mM), Na3PO4 (2.5 mM), NaF (5 mM), Triton X-100 (1%), and glycerol (10%) buffer, supplemented with protease inhibitor cocktail (Roche), phosphatase inhibitor cocktail and 1% PMSF (Sigma Aldrich). The following antibodies were used: Akt, pAkt^S473^, Btk, pBtk^Y223^, Syk, pSyk^Y525/526^, Phospho-Zap-70 ^Y319^ /Syk ^Y352^ , Phospho-p44/42 MAPK^T202/Y204^, Bcl-xL, Bcl-2, Mcl-1, p65, p100/p52, IκBα, GAPDH, β-Actin, and horseradish peroxidase conjugated anti-rabbit and anti-mouse antibodies, all from Cell Signaling Technologies. For BCR crosslinking, cells were incubated with drugs for 1 hour, stimulated with 5 µg/mL anti-IgM (Southern Biotech), washed and subjected to lysis and immunoblotting.

*NF-κB ELISA-based assay*

Activation of NF-κB family constituents was quantified using the TransAM NF-κB Family Kit (Active Motif). The manufacturer’s protocol was followed. Briefly, frozen viable splenocytes were thawed and live cells were isolated using the Ficoll-Hypaque technique. Nuclei were extracted using sonication with a Fisherbrand model CL-334 sonicator and model FB505 system programmed for 6 cycles of 3 second pulses with 9 second breaks. Protein was harvested from the nuclear fraction and assayed at 10 µg per sample.

*Quantitative PCR*

Total RNA was extracted using the ENZA Total RNA Kit I (Omega Bio-Tek). cDNA was synthesized from 500 ng of RNA using qScript cDNA Supermix (QuantaBio). cDNA was mixed with gene-specific probes and PerfeCTa FastMix II as recommended by the manufacturer (Quantabio) then Quantitative real-time PCR (RT-PCR) was performed using a QuantStudio 7 Flex (Applied Biosys-tems). The following probes were used: *BIM*, Hs00708019_s1; *BCL2L1*, Hs99999147_m1; *BAX*, Hs00180269_m1. Amplification of the sequence of interest was compared with a reference probe (*GAPDH*, Hs02758991_g1, all from Life Technologies). All samples were prepared in technical triplicate. The comparative Ct method was used for relative quantitation (2^-Δ ΔCt^, where ΔΔC_t_ = ΔC_tp_ - ΔC_tK_ ; where P = probe and K = reference sample).

*RNA-seq*

Total RNA was isolated using the E.Z.N.A. HP Total RNA Isolation Kit (Omega Bio-tek). RNA concentration was determined using the Qubit RNA HS Assay Kit (Invitrogen). 2500ng of Total RNA (100 ng/µL) per sample was submitted to Novogene Corporation. RNA integrity was determined using the 2100 Bioanalyzer Instrument (Agilent). Library preparation and sequencing was carried out by Novogene Corporation. Samples were sequenced 12 per lane on a NovaSeq 6000 (Illumina). Trimming of Fastq files was performed with Trimmomatic, QC was performed with FASTQC, mapping to GRCh38 was performed with STAR, DGE was performed using edgeR filtering for genes that were expressed in at least one sample. We defined up/downregulated genes by statistical significance (Padj <0.05) and effect size (log_­2_fc ≥0.58/≤-0.58). Gene set enrichment analysis was carried out using gsea2-2.24. Full results are available at:

https://www.ncbi.nlm.nih.gov/geo/query/acc.cgi?acc=GSE173353

*Seahorse MitoStress Test Assay*

The Seahorse XF Cell Mito Stress Test Kit (CAT# 103015-100, Seahorse Bioscience) was used to probe the cellular rate of oxidative phosphorylation (OxPhos) and glycolysis. Briefly, XFe96 Cell Culture Microplates (Seahorse Bioscience) were coated with Poly-D-Lysine (Thermo Fisher Scientific), then were seeded with 8x10^4^ cells per well. Plates were centrifuged at 300g for 5 minutes without breaks. Cells were then incubated for 30 minutes at 37°C with no CO_2_ supplemented. Oxygen consumption rate and extracellular acidification were measured using an XFe96 Extracellular Flux Analyzer (Seahorse Bioscience). Oligomycin (1.5 µM), FCCP (1 µM), and Rotenone/Antimycin A (0.5 µM) were injected at the recommended timepoints.

*Functional analysis of mitochondria*

Mitochondrial depolarization was quantified using the JC-1 MitoMP Detection Kit (Dojindo Molecular Technologies). After 24-hour incubation with drugs, cells were stained with JC-1 dye at 4 µM concentration for 30 min, washed, resuspended in 1x imaging solution and immediately analyzed by flow cytometry. Red indicates healthy polarized mitochondria (JC-1 aggregates), whereas green indicates loss of membrane potential (JC-1 monomers).

To quantify mitochondrial mass, 1x10^4^ cells/condition were treated with drugs for 24 hours in 48-well plates, washed and incubated in 20 nM MitoBright Green (Dojindo Molecular Technologies) and Live/Dead Aqua (Molecular Probes) for 30 min. Cells were washed and analyzed by flow cytometry. MitoBright green MFI was quantified, with lower intensity staining indicating a decrease in mitochondrial mass.

To quantify mitophagy, cells were incubated for 30 minutes in serum-free RPMI medium with 100 nM mitophagy Dye (Mitophagy Detection Kit, Dojindo Molecular Technologies) and treated with drugs for 24 hours. Next, cells were washed and incubated in serum-free medium with 1 µM lysodye for 30 minutes. 2x10^5^ cells suspended in 100 µL medium were seeded in poly-lysine-coated 24-well black imaging plates (Ibidi). Plates were centrifuged at 300 g for 5 minutes with no breaks and analyzed by fluorescent microscopy on a Zeiss LSM 700 Confocal Microscope (Carl Zeiss AG). Location of lysosomes and damaged mitochondria was inferred by the intensity of lysodye (488 nm, 550 nm) and mitophagy dye (561 nm, 570-700 nm), respectively. Fluorescence intensity was calculated in >20 cells which were randomly selected in each high-power microscopy field.

*Reactive Oxygen Species (ROS) Measurement*

The DCFDA / H2DCFDA - Cellular ROS Assay Kit (Abcam) was used to detect intracellular ROS. Briefly, cells were incubated for 30 minutes in PBS containing the DCFDA dye, washed and seeded at 1x10^5^ cells per well in a 96-well plate. Cells were treated with drug for 24 hours then harvested and immediately analyzed by flow cytometry. The proton gradient uncoupler carbonyl cyanide m-chlorophenyl hydrazone (CCCP, 10 µM) was used as a positive control for ROS generation.

*Genome-wide loss-of-function CRISPR-Cas9 Screen*

The previously described protocol was followed.^2^ Briefly, Cas9-expressing OCI-LY3 cells were transduced with the Y. Kosuke human genome-wide library, an sgRNA library targeting 18,010 different genes, averaging 5 sgRNA per gene (Addgene, CAT# 67989).^3^ Cells were then treated with either CG-806 (1 µM) or DMSO for 7 days. Genomic DNA was extracted and barcodes corresponding to sgRNAs were PCR amplified and sequenced. Reads were first trimmed using cutadapt (v2.3) (1) and aligned to the sgRNA library using bowtie2 (v2.3.5.1) (2) limited to unique matches allowing no mismatches.  Reads were counted using MAGeCK count (v0.5.8) (3).  An edgeR (v3.32.0) (4) analysis pipeline was utilized to compute the differential abundance between CG-806 and DMSO per sgRNA.  For each treatment versus DMSO comparison, only sgRNAs with ≥100 reads in at least half the samples were included in statistical analysis.  We defined a gene-level summary by first ordering each sgRNA within a gene by P-value and recording the log fold change and P-value of the ‘mid’ sgRNA.  The statistic sign(lfc) * -log10(P-value) of the gene level values were then used in pathway analysis. Differentially enriched gene sets were identified using webgestalt software referencing the Wikipathway cancer database.^2^

*Genome editing by RNP electroporation*

Genome editing was carried out in OCI-LY3 cell lines using RNP electroporation with the Lonza Amaxa Nucleofector II System (Lonza, Basel, Switzerland), 4 μM (1:3, Cas9:sgRNA) Alt-R® (Integrated DNA Technologies, Inc) Cas9 RNP complex and 4 μM Alt-R® Cas9 Electroporation Enhancer (Integrated DNA Technologies, Inc), as described.^4^ Chemically synthesized guide RNAs (Synthego, CA, USA) were prepared as recommended by the manufacturer, then purified Cas9 protein was added for a 10-minute incubation at room temperature to promote the formation of RNP complexes (IDT). Cells were suspended in PBS (1.5x10^5^, 20 μL) then RNP complexes were added (5 μL). Electroporation was carried out as detailed in the standard Amaxa protocol V-001. Protein was harvested 6 days after electroporation. The targeting sequences were: NT sgRNA: GAGATATCAATCCTCCCGC; *BAX*_1: AGUUUCAUCCAGGAUCGAGC; *BAX*_2: AGUAGAAAAGGGCGACAACC; *NFKBIA* sgRNA pool: CACCUGGCGGAUCACUUCCA, UGCCUUUUCUUCAUGGAUGA, GGCCAUCAUCCAUGAAGAAA.

*Functional drug screen*

Functional drug screening was carried out as previously described.^5, 6^ The screen included 189 small-molecule inhibitors purchased from LC Laboratories and Selleck Chemicals which were dissolved in DMSO and stored at −80 °C. For single agent tests (i.e., venetoclax), inhibitors were aliquoted in 384-well plates in a seven-point concentration series (10–0.0137 μM), while inhibitors in dual agent tests (ie. olaparib – venetoclax) were prepared in an seven-point concentration series at fixed molar ratio with concentrations identical to the single agents. Drug-treated plates were stored at −20 °C. Cells were seeded at 1x10^4^ per well and treated with CG-806 (1 μM) or vehicle control. Following a 72-hour incubation at 37 °C in 5% CO_2_, cell viability was analyzed using the MTS reagent as described above. IC_50_ was calculated and compared between the DMSO and CG-806- treated plates. A 7-by-7 matrix MTS assay was performed to validate findings, and synergy score was calculated in R using the highest single agent (HSA) reference model. In this screen, proprietary drugs are identified by their target (i.e., BCL2/Xi, BETi).

**Supplemental Figure Legends.**

**Supplemental Figure 1.**

DLBCL cell lines were treated with the indicated doses of CG-806 for 72 hours. Cell proliferation was assessed using a colorimetric tetrazolium-based assay. Mean ± SEM is shown. *p<0.05 and **p<0.01 vs. untreated control.

**Supplemental Figure 2.**

Immunoblots corresponding to Figure 1C.

**Supplemental Figure 3.**

Immunoblots corresponding to Figure 1D-E.

**Supplemental Figure 4.**

(A) Cell lines were stained with DCFDA then treated with drugs for 24 h. MFI was measured at the indicated timepoints. (B) Jeko-1 and VAL cells were treated with 1 µM CG-806 vs. control for 24 hours and stained with Mtphagy dye and Lysodye. Live cells were imaged with confocal microscopy. A representative image is shown. White arrows point to the mitophagy puncta. MFI was quantified, and co-localization was measured and calculated using Zen software. Data are presented as mean ± SEM. *, p<0.05 and **, p<0.01 vs. untreated control.

**Supplemental Figure 5.**

Immunoblots corresponding to Figure 3C.

**Supplemental Figure 6.**

Volcano plot of fold change vs. p-value of genes in RNA-seq (CG-806 308mg/kg vs control). Significantly differentially regulated genes (log_­2_fc ≥0.58/≤-0.58, Padj < 0.05) are shown in red (upregulated) and blue (downregulated).

**Supplemental Figure 7.**

Immunoblots corresponding to Figure 5C-D.

**Supplemental Figure 8.**

(A) Visualization of calculated synergy map based on dose response matrix. U2932 cells were treated with CG-806, venetoclax, or a combination of the two in an 7X7 matrix as indicated for 48 hours. (B) MCL cells were treated with venetoclax at the indicated concentrations for 48 hours. Cell proliferation was measured using a colorimetric tetrazolium-based assay; % viable cells were normalized to DMSO-treated control. Mean ± SEM is shown.

**Supplemental References**

1. Paiva C, Rowland TA, Sreekantham B, Godbersen C, Best SR, Kaur P*, et al.* SYK inhibition thwarts the BAFF - B-cell receptor crosstalk and thereby antagonizes Mcl-1 in chronic lymphocytic leukemia. *Haematologica* 2017 Nov; **102**(11)**:** 1890-1900.

2. Nechiporuk T, Kurtz SE, Nikolova O, Liu T, Jones CL, D'Alessandro A*, et al.* The TP53 Apoptotic Network Is a Primary Mediator of Resistance to BCL2 Inhibition in AML Cells. *Cancer Discov* 2019 Jul; **9**(7)**:** 910-925.

3. Tzelepis K, Koike-Yusa H, De Braekeleer E, Li Y, Metzakopian E, Dovey OM*, et al.* A CRISPR Dropout Screen Identifies Genetic Vulnerabilities and Therapeutic Targets in Acute Myeloid Leukemia. *Cell Rep* 2016 Oct 18; **17**(4)**:** 1193-1205.

4. Vakulskas CA, Dever DP, Rettig GR, Turk R, Jacobi AM, Collingwood MA*, et al.* A high-fidelity Cas9 mutant delivered as a ribonucleoprotein complex enables efficient gene editing in human hematopoietic stem and progenitor cells. *Nat Med* 2018 Aug; **24**(8)**:** 1216-1224.

5. Tyner JW, Tognon CE, Bottomly D, Wilmot B, Kurtz SE, Savage SL*, et al.* Functional genomic landscape of acute myeloid leukaemia. *Nature* 2018 Oct; **562**(7728)**:** 526-531.

6. Eide CA, Kurtz SE, Kaempf A, Long N, Agarwal A, Tognon CE*, et al.* Simultaneous kinase inhibition with ibrutinib and BCL2 inhibition with venetoclax offers a therapeutic strategy for acute myeloid leukemia. *Leukemia* 2020 Sep; **34**(9)**:** 2342-2353.
