# Supplementary material for: Dual BTK/SYK inhibition with CG-806 (luxeptinib) disrupts B-cell receptor and Bcl-2 signaling networks in mantle cell lymphoma
Source: Cell Death Dis. 2022 Mar 16;13(3):246. doi: 10.1038/s41419-022-04684-1 (PMC8927405; doi:10.1038/s41419-022-04684-1)
Supplement: Supplementary file 3 — Authorship Change [file 41419_2022_4684_MOESM3_ESM.pdf]

Alexey Danilov MD PhD

From: Daniel Bottomly <bottomly@ohsu.edu>
Sent: Thursday, February 17, 2022 3:14 PM
To: Alexey Danilov MD PhD; Jeffrey Tyner; Steve Kurtz; Shannon McWeeney; Tamilla Nechiporuk
Subject: Re: CDDIS-21-2513

Hi:

Yes, I agree

Dan

From: Alexey Danilov MD PhD <adanilov@coh.org>
Date: Thursday, February 17, 2022 at 2:59 PM
To: Jeffrey Tyner <tynerj@ohsu.edu>, Steve Kurtz <kurtzs@ohsu.edu>, Shannon McWeeney <mcweeney@ohsu.edu>, Daniel Bottomly <bottomly@ohsu.edu>, Tamilla Nechiporuk <nechipot@ohsu.edu>
Subject: [EXTERNAL] CDDIS-21-2513

Dear co-authors,

Will you please respond to this e-mail "Yes, I agree" if you agree with the following change

In reference to manuscript CDDIS-21-2513 **Dual BTK/SYK inhibition with CG-806 (luxeptinib) disrupts B-cell receptor and Bcl-2 signaling networks in mantle cell lymphoma** which has been provisionally accepted by Cell Death and Disease. Following revisions, we are planning to modify authorship:

1) Tingting Liu is co-first author
2) Carly Roleder is added to the author list

As a result, the change is as follows:

ORIGINAL author list:

Elana Thieme[1], Nur Bruss[2], Vi Lam[1], Xiaoguang Wang[1], Tamilla Nechiporuk[2,3], Geoffrey Shouse[1], Olga V. Danilova[1], Daniel Bottomly[2,4], Shannon K. McWeeney[2,4,5], Jeffrey W. Tyner[2,3,6], Stephen E. Kurtz[2,3], Alexey V. Danilov[1,2]* and Tingting Liu[1]*

* - A.V.D. and T.L. contributed equally to this work

UPDATED author list:

Elana Thieme[1*], Tingting Liu[1]*, Nur Bruss[2], Carly Roleder[1], Vi Lam[1], Xiaoguang Wang[1], Tamilla Nechiporuk[2,3], Geoffrey Shouse[1], Olga V. Danilova[1], Daniel Bottomly[2,4], Shannon K. McWeeney[2,4,5], Jeffrey W. Tyner[2,3,6], Stephen E. Kurtz[2,3], Alexey V. Danilov[1,2]

* - E.T. and T.L. contributed equally to this work

-SECURITY/CONFIDENTIALITY WARNING-

This message and any attachments are intended solely for the individual or entity to which they are addressed. This communication may contain information that is privileged, confidential, or exempt from disclosure under applicable law (e.g., personal health information, research data, financial information). Because this e-mail has been sent without encryption, individuals other than the intended recipient may be able to view the information, forward it to others or tamper with the information without the knowledge or consent of the sender. If you are not the intended recipient, or the employee or person responsible for delivering the message to the intended recipient, any dissemination, distribution or copying of the communication is strictly prohibited. If you received the communication in error, please notify the sender immediately by replying to this message and deleting the message and any accompanying files from your system. If, due to the security risks, you do not wish to receive further communications via e-mail, please reply to this message and inform the sender that you do not wish to receive further e-mail from the sender. (LCP301)

Alexey Danilov MD PhD

From: Steve Kurtz <kurtzs@ohsu.edu>
Sent: Thursday, February 17, 2022 3:21 PM
To: Alexey Danilov MD PhD; Jeffrey Tyner; Shannon McWeeney; Daniel Bottomly; Tamilla Nechiporuk
Subject: Re: CDDIS-21-2513

Yes,

I agree. -Steve

From: Alexey Danilov MD PhD <adanilov@coh.org>
Date: Thursday, February 17, 2022 at 2:59 PM
To: Jeffrey Tyner <tynerj@ohsu.edu>, Steve Kurtz <kurtzs@ohsu.edu>, Shannon McWeeney <mcweeney@ohsu.edu>, Daniel Bottomly <bottomly@ohsu.edu>, Tamilla Nechiporuk <nechipot@ohsu.edu>
Subject: [EXTERNAL] CDDIS-21-2513

Dear co-authors,

Will you please respond to this e-mail "Yes, I agree" if you agree with the following change

In reference to manuscript CDDIS-21-2513 **Dual BTK/SYK inhibition with CG-806 (luxeptinib) disrupts B-cell receptor and Bcl-2 signaling networks in mantle cell lymphoma** which has been provisionally accepted by Cell Death and Disease. Following revisions, we are planning to modify authorship:

1) Tingting Liu is co-first author
2) Carly Roleder is added to the author list

As a result, the change is as follows:

ORIGINAL author list:

Elana Thieme[1], Nur Bruss[2], Vi Lam[1], Xiaoguang Wang[1], Tamilla Nechiporuk[2,3], Geoffrey Shouse[1], Olga V. Danilova[1], Daniel Bottomly[2,4], Shannon K. McWeeney[2,4,5], Jeffrey W. Tyner[2,3,6], Stephen E. Kurtz[2,3], Alexey V. Danilov[1,2]* and Tingting Liu[1]*

* - A.V.D. and T.L. contributed equally to this work

UPDATED author list:

Elana Thieme[1*], Tingting Liu[1]*, Nur Bruss[2], Carly Roleder[1], Vi Lam[1], Xiaoguang Wang[1], Tamilla Nechiporuk[2,3], Geoffrey Shouse[1], Olga V. Danilova[1], Daniel Bottomly[2,4], Shannon K. McWeeney[2,4,5], Jeffrey W. Tyner[2,3,6], Stephen E. Kurtz[2,3], Alexey V. Danilov[1,2]

* - E.T. and T.L. contributed equally to this work

-SECURITY/CONFIDENTIALITY WARNING-

This message and any attachments are intended solely for the individual or entity to which they are addressed. This communication may contain information that is privileged, confidential, or exempt from disclosure under applicable law (e.g., personal health information, research data, financial information). Because this e-mail has been sent without encryption, individuals other than the intended recipient may be able to view the information, forward it to others or tamper with the information without the knowledge or consent of the sender. If you are not the intended recipient, or the employee or person responsible for delivering the message to the intended recipient, any dissemination, distribution or copying of the communication is strictly prohibited. If you received the communication in error, please notify the sender immediately by replying to this message and deleting the message and any accompanying files from your system. If, due to the security risks, you do not wish to receive further communications via e-mail, please reply to this message and inform the sender that you do not wish to receive further e-mail from the sender. (LCP301)

Alexey Danilov MD PhD

From: Shannon McWeeney <mcweeney@ohsu.edu>
Sent: Friday, February 18, 2022 7:55 AM
To: Alexey Danilov MD PhD; Jeffrey Tyner; Steve Kurtz; Daniel Bottomly; Tamilla Nechiporuk
Subject: Re: CDDIS-21-2513

Yes I agree

Shannon McWeeney, Ph.D.
Professor, Biostatistics & Bioinformatics
Head, Division of Bioinformatics & Computational Biology
Chief Data Officer, OHSU Knight Cancer Institute
Director, Medical Bioinformatics, OHSU Knight SMMART Program
Co-Director, Informatics, Oregon Clinical and Translational Research Institute
Web: http://www.biodevlab.org

Administrative Assistant: Nicole Durchanek
Email: durchane@ohsu.edu
Phone: 503-418-9667

Please note: given my research, mentoring and leadership responsibilities, there can be multiple holds on my calendar. Nicole is essential to my high functioning abilities so please be patient when she is scheduling when she needs time to confirm appts.

From: Alexey Danilov MD PhD <adanilov@coh.org>
Date: Thursday, February 17, 2022 at 2:59 PM
To: Jeffrey Tyner <tynerj@ohsu.edu>, Steve Kurtz <kurtzs@ohsu.edu>, Shannon McWeeney <mcweeney@ohsu.edu>, Daniel Bottomly <bottomly@ohsu.edu>, Tamilla Nechiporuk <nechipot@ohsu.edu>
Subject: [EXTERNAL] CDDIS-21-2513

Dear co-authors,

Will you please respond to this e-mail "Yes, I agree" if you agree with the following change

In reference to manuscript CDDIS-21-2513 **Dual BTK/SYK inhibition with CG-806 (luxeptinib) disrupts B-cell receptor and Bcl-2 signaling networks in mantle cell lymphoma** which has been provisionally accepted by Cell Death and Disease. Following revisions, we are planning to modify authorship:

1) Tingting Liu is co-first author

2) Carly Roleder is added to the author list

As a result, the change is as follows:

ORIGINAL author list:

Elana Thieme[1], Nur Bruss[2], Vi Lam[1], Xiaoguang Wang[1], Tamilla Nechiporuk[2,3], Geoffrey Shouse[1], Olga V. Danilova[1], Daniel Bottomly[2,4], Shannon K. McWeeney[2,4,5], Jeffrey W. Tyner[2,3,6], Stephen E. Kurtz[2,3], Alexey V. Danilov[1,2]* and Tingting Liu[1]*

* - A.V.D. and T.L. contributed equally to this work

UPDATED author list:

Elana Thieme[1*], Tingting Liu[1]*, Nur Bruss[2], Carly Roleder[1], Vi Lam[1], Xiaoguang Wang[1], Tamilla Nechiporuk[2,3], Geoffrey Shouse[1], Olga V. Danilova[1], Daniel Bottomly[2,4], Shannon K. McWeeney[2,4,5], Jeffrey W. Tyner[2,3,6], Stephen E. Kurtz[2,3], Alexey V. Danilov[1,2]

* - E.T. and T.L. contributed equally to this work

-SECURITY/CONFIDENTIALITY WARNING-

This message and any attachments are intended solely for the individual or entity to which they are addressed. This communication may contain information that is privileged, confidential, or exempt from disclosure under applicable law (e.g., personal health information, research data, financial information). Because this e-mail has been sent without encryption, individuals other than the intended recipient may be able to view the information, forward it to others or tamper with the information without the knowledge or consent of the sender. If you are not the intended recipient, or the employee or person responsible for delivering the message to the intended recipient, any dissemination, distribution or copying of the communication is strictly prohibited. If you received the communication in error, please notify the sender immediately by replying to this message and deleting the message and any accompanying files from your system. If, due to the security risks, you do not wish to receive further communications via e-mail, please reply to this message and inform the sender that you do not wish to receive further e-mail from the sender. (LCP301)

Alexey Danilov MD PhD

From: Xiao Wang
Sent: Thursday, February 17, 2022 2:22 PM
To: Alexey Danilov MD PhD
Subject: RE: CDDIS-21-2513

Yes, I agree. Thanks.

Best,

Xiao

From: Alexey Danilov MD PhD <adanilov@coh.org>
Sent: Thursday, February 17, 2022 2:19 PM
To: Elana Thieme <ethieme@coh.org>; Nur Bruss <bruss@ohsu.edu>; Tingting Liu <tliu@coh.org>; Vi Lam <vilam@coh.org>; Xiao Wang <xiwang@coh.org>
Subject: CDDIS-21-2513

Dear co-authors,

Will you please respond to this e-mail "Yes, I agree" if you agree with the following change

In reference to manuscript CDDIS-21-2513 **Dual BTK/SYK inhibition with CG-806 (luxeptinib) disrupts B-cell receptor and Bcl-2 signaling networks in mantle cell lymphoma** which has been provisionally accepted by Cell Death and Disease. Following revisions, we are planning to modify authorship:

1) Tingting Liu is co-first author
2) Carly Roleder is added to the author list

As a result, the change is as follows:

ORIGINAL author list:

Elana Thieme[1], Nur Bruss[2], Vi Lam[1], Xiaoguang Wang[1], Tamilla Nechiporuk[2,3], Geoffrey Shouse[1], Olga V. Danilova[1], Daniel Bottomly[2,4], Shannon K. McWeeney[2,4,5], Jeffrey W. Tyner[2,3,6], Stephen E. Kurtz[2,3], Alexey V. Danilov[1,2]* and Tingting Liu[1]*

* - A.V.D. and T.L. contributed equally to this work

UPDATED author list:

Elana Thieme[1*], Tingting Liu[1]*, Nur Bruss[2], Carly Roleder[1], Vi Lam[1], Xiaoguang Wang[1], Tamilla Nechiporuk[2,3], Geoffrey Shouse[1], Olga V. Danilova[1], Daniel Bottomly[2,4], Shannon K. McWeeney[2,4,5], Jeffrey W. Tyner[2,3,6], Stephen E. Kurtz[2,3], Alexey V. Danilov[1,2]

* - E.T. and T.L. contributed equally to this work

Alexey Danilov MD PhD

From: Elana Thieme
Sent: Thursday, February 17, 2022 2:23 PM
To: Alexey Danilov MD PhD
Subject: Re: CDDIS-21-2513

Yes, I agree.

Elana Thieme
Research Associate I Toni Stephenson Lymphoma Center
1500 E Duarte Rd, Duarte, CA 91010 (Bldg #158 room #1022)
ethieme@coh.org
Phone 626-218-8833

From: Alexey Danilov MD PhD <adanilov@coh.org>
Sent: Thursday, February 17, 2022 2:19 PM
To: Elana Thieme <ethieme@coh.org>; Nur Bruss <bruss@ohsu.edu>; Tingting Liu <tliu@coh.org>; Vi Lam <vilam@coh.org>; Xiao Wang <xiwang@coh.org>
Subject: CDDIS-21-2513

Dear co-authors,

Will you please respond to this e-mail "Yes, I agree" if you agree with the following change

In reference to manuscript CDDIS-21-2513 **Dual BTK/SYK inhibition with CG-806 (luxeptinib) disrupts B-cell receptor and Bcl-2 signaling networks in mantle cell lymphoma** which has been provisionally accepted by Cell Death and Disease. Following revisions, we are planning to modify authorship:

1. Tingting Liu is co-first author
2. Carly Roleder is added to the author list

As a result, the change is as follows:

ORIGINAL author list:

Elana Thieme[1], Nur Bruss[2], Vi Lam[1], Xiaoguang Wang[1], Tamilla Nechiporuk[2,3], Geoffrey Shouse[1], Olga V. Danilova[1], Daniel Bottomly[2,4], Shannon K. McWeeney[2,4,5], Jeffrey W. Tyner[2,3,6], Stephen E. Kurtz[2,3], Alexey V. Danilov[1,2]* and Tingting Liu[1]*

* - A.V.D. and T.L. contributed equally to this work

UPDATED author list:

Elana Thieme[1*], Tingting Liu[1*], Nur Bruss[2], Carly Roleder[1], Vi Lam[1], Xiaoguang Wang[1], Tamilla Nechiporuk[2,3], Geoffrey Shouse[1], Olga V. Danilova[1], Daniel Bottomly[2,4], Shannon K. McWeeney[2,4,5], Jeffrey W. Tyner[2,3,6], Stephen E. Kurtz[2,3], Alexey V. Danilov[1,2]

* - E.T. and T.L. contributed equally to this work

Alexey Danilov MD PhD

From: Tingting Liu
Sent: Thursday, February 17, 2022 2:29 PM
To: Alexey Danilov MD PhD
Subject: Re: CDDIS-21-2513

I agree with the changes.
Tingting

Get Outlook for iOS

From: Alexey Danilov MD PhD <adanilov@coh.org>
Sent: Thursday, February 17, 2022 2:19:15 PM
To: Elana Thieme <ethieme@coh.org>; Nur Bruss <bruss@ohsu.edu>; Tingting Liu <tliu@coh.org>; Vi Lam <vilam@coh.org>; Xiao Wang <xiwang@coh.org>
Subject: CDDIS-21-2513

Dear co-authors,

Will you please respond to this e-mail "Yes, I agree" if you agree with the following change

In reference to manuscript CDDIS-21-2513 **Dual BTK/SYK inhibition with CG-806 (luxeptinib) disrupts B-cell receptor and Bcl-2 signaling networks in mantle cell lymphoma** which has been provisionally accepted by Cell Death and Disease. Following revisions, we are planning to modify authorship:

1. Tingting Liu is co-first author
2. Carly Roleder is added to the author list

As a result, the change is as follows:

ORIGINAL author list:

Elana Thieme[1], Nur Bruss[2], Vi Lam[1], Xiaoguang Wang[1], Tamilla Nechiporuk[2,3], Geoffrey Shouse[1], Olga V. Danilova[1], Daniel Bottomly[2,4], Shannon K. McWeeney[2,4,5], Jeffrey W. Tyner[2,3,6], Stephen E. Kurtz[2,3], Alexey V. Danilov[1,2]* and Tingting Liu[1]*

* - A.V.D. and T.L. contributed equally to this work

UPDATED author list:

Elana Thieme[1*], Tingting Liu[1]*, Nur Bruss[2], Carly Roleder[1], Vi Lam[1], Xiaoguang Wang[1], Tamilla Nechiporuk[2,3], Geoffrey Shouse[1], Olga V. Danilova[1], Daniel Bottomly[2,4], Shannon K. McWeeney[2,4,5], Jeffrey W. Tyner[2,3,6], Stephen E. Kurtz[2,3], Alexey V. Danilov[1,2]

* - E.T. and T.L. contributed equally to this work

Alexey Danilov MD PhD

From: Geoffrey Shouse DO PhD
Sent: Thursday, February 17, 2022 3:01 PM
To: Alexey Danilov MD PhD
Subject: Re: CDDIS-21-2513

Yes I agree

Thanks,

Geoffrey Shouse D.O., Ph.D.
Assistant Clinical Professor
Hematology and Hematopoietic Cell Transplant

From: Alexey Danilov MD PhD <adanilov@coh.org>
Sent: Thursday, February 17, 2022 2:59:47 PM
To: Geoffrey Shouse DO PhD <gshouse@coh.org>; Olga Danilova MD <odanilova@coh.org>
Subject: CDDIS-21-2513

Dear co-authors,

Will you please respond to this e-mail "Yes, I agree" if you agree with the following change

In reference to manuscript CDDIS-21-2513 **Dual BTK/SYK inhibition with CG-806 (luxeptinib) disrupts B-cell receptor and Bcl-2 signaling networks in mantle cell lymphoma** which has been provisionally accepted by Cell Death and Disease. Following revisions, we are planning to modify authorship:

1. Tingting Liu is co-first author
2. Carly Roleder is added to the author list

As a result, the change is as follows:

ORIGINAL author list:

Elana Thieme[1], Nur Bruss[2], Vi Lam[1], Xiaoguang Wang[1], Tamilla Nechiporuk[2,3], Geoffrey Shouse[1], Olga V. Danilova[1], Daniel Bottomly[2,4], Shannon K. McWeeney[2,4,5], Jeffrey W. Tyner[2,3,6], Stephen E. Kurtz[2,3], Alexey V. Danilov[1,2]* and Tingting Liu[1]*

* - A.V.D. and T.L. contributed equally to this work

UPDATED author list:

Elana Thieme[1*], Tingting Liu[1]*, Nur Bruss[2], Carly Roleder[1], Vi Lam[1], Xiaoguang Wang[1], Tamilla Nechiporuk[2,3], Geoffrey Shouse[1], Olga V. Danilova[1], Daniel Bottomly[2,4], Shannon K. McWeeney[2,4,5], Jeffrey W. Tyner[2,3,6], Stephen E. Kurtz[2,3], Alexey V. Danilov[1,2]

* - E.T. and T.L. contributed equally to this work

From: Vi Lam
Sent: Thursday, February 17, 2022 3:07 PM
To: Alexey Danilov MD PhD
Subject: RE: CDDIS-21-2513

Yes, I agree

Vi

From: Alexey Danilov MD PhD <adanilov@coh.org>
Sent: Thursday, February 17, 2022 2:19 PM
To: Elana Thieme <ethieme@coh.org>; Nur Bruss <bruss@ohsu.edu>; Tingting Liu <tliu@coh.org>; Vi Lam <vilam@coh.org>; Xiao Wang <xiwang@coh.org>
Subject: CDDIS-21-2513

Dear co-authors,

Will you please respond to this e-mail "Yes, I agree" if you agree with the following change

In reference to manuscript CDDIS-21-2513 **Dual BTK/SYK inhibition with CG-806 (luxeptinib) disrupts B-cell receptor and Bcl-2 signaling networks in mantle cell lymphoma** which has been provisionally accepted by Cell Death and Disease. Following revisions, we are planning to modify authorship:

1) Tingting Liu is co-first author
2) Carly Roleder is added to the author list

As a result, the change is as follows:

ORIGINAL author list:

Elana Thieme[1], Nur Bruss[2], Vi Lam[1], Xiaoguang Wang[1], Tamilla Nechiporuk[2,3], Geoffrey Shouse[1], Olga V. Danilova[1], Daniel Bottomly[2,4], Shannon K. McWeeney[2,4,5], Jeffrey W. Tyner[2,3,6], Stephen E. Kurtz[2,3], Alexey V. Danilov[1,2]* and Tingting Liu[1]*

* - A.V.D. and T.L. contributed equally to this work

UPDATED author list:

Elana Thieme[1*], Tingting Liu[1]*, Nur Bruss[2], Carly Roleder[1], Vi Lam[1], Xiaoguang Wang[1], Tamilla Nechiporuk[2,3], Geoffrey Shouse[1], Olga V. Danilova[1], Daniel Bottomly[2,4], Shannon K. McWeeney[2,4,5], Jeffrey W. Tyner[2,3,6], Stephen E. Kurtz[2,3], Alexey V. Danilov[1,2]

* - E.T. and T.L. contributed equally to this work

Alexey Danilov MD PhD

From: Carly Roleder
Sent: Thursday, February 17, 2022 3:49 PM
To: Alexey Danilov MD PhD
Subject: RE: CDDIS-21-2513

Yes, I agree

-Carly Roleder

From: Alexey Danilov MD PhD <adanilov@coh.org>
Sent: Thursday, February 17, 2022 3:00 PM
To: Carly Roleder <croleder@coh.org>
Subject: CDDIS-21-2513

Dear Carly,

Will you please respond to this e-mail "Yes, I agree" if you agree with the following change

In reference to manuscript CDDIS-21-2513 **Dual BTK/SYK inhibition with CG-806 (luxeptinib) disrupts B-cell receptor and Bcl-2 signaling networks in mantle cell lymphoma** which has been provisionally accepted by Cell Death and Disease. Following revisions, we are planning to modify authorship:

1) Tingting Liu is co-first author
2) Carly Roleder is added to the author list

As a result, the change is as follows:

ORIGINAL author list:

Elana Thieme[1], Nur Bruss[2], Vi Lam[1], Xiaoguang Wang[1], Tamilla Nechiporuk[2,3], Geoffrey Shouse[1], Olga V. Danilova[1], Daniel Bottomly[2,4], Shannon K. McWeeney[2,4,5], Jeffrey W. Tyner[2,3,6], Stephen E. Kurtz[2,3], Alexey V. Danilov[1,2]* and Tingting Liu[1]*

* - A.V.D. and T.L. contributed equally to this work

UPDATED author list:

Elana Thieme[1*], Tingting Liu[1]*, Nur Bruss[2], Carly Roleder[1], Vi Lam[1], Xiaoguang Wang[1], Tamilla Nechiporuk[2,3], Geoffrey Shouse[1], Olga V. Danilova[1], Daniel Bottomly[2,4], Shannon K. McWeeney[2,4,5], Jeffrey W. Tyner[2,3,6], Stephen E. Kurtz[2,3], Alexey V. Danilov[1,2]

* - E.T. and T.L. contributed equally to this work

Alexey Danilov MD PhD

From: Jeffrey Tyner <tynerj@ohsu.edu>
Sent: Thursday, February 17, 2022 4:02 PM
To: Alexey Danilov MD PhD; Steve Kurtz; Shannon McWeeney; Daniel Bottomly; Tamilla Nechiporuk
Subject: Re: CDDIS-21-2513

Yes, I agree

From: Alexey Danilov MD PhD <adanilov@coh.org>
Sent: Thursday, February 17, 2022 2:59:08 PM
To: Jeffrey Tyner; Steve Kurtz; Shannon McWeeney; Daniel Bottomly; Tamilla Nechiporuk
Subject: [EXTERNAL] CDDIS-21-2513

Dear co-authors,

Will you please respond to this e-mail "Yes, I agree" if you agree with the following change

In reference to manuscript CDDIS-21-2513 **Dual BTK/SYK inhibition with CG-806 (luxeptinib) disrupts B-cell receptor and Bcl-2 signaling networks in mantle cell lymphoma** which has been provisionally accepted by Cell Death and Disease. Following revisions, we are planning to modify authorship:

1) Tingting Liu is co-first author

2) Carly Roleder is added to the author list

As a result, the change is as follows:

ORIGINAL author list:

Elana Thieme[1], Nur Bruss[2], Vi Lam[1], Xiaoguang Wang[1], Tamilla Nechiporuk[2,3], Geoffrey Shouse[1], Olga V. Danilova[1], Daniel Bottomly[2,4], Shannon K. McWeeney[2,4,5], Jeffrey W. Tyner[2,3,6], Stephen E. Kurtz[2,3], Alexey V. Danilov[1,2]* and Tingting Liu[1]*

* - A.V.D. and T.L. contributed equally to this work

UPDATED author list:

Elana Thieme[1*], Tingting Liu[1]*, Nur Bruss[2], Carly Roleder[1], Vi Lam[1], Xiaoguang Wang[1], Tamilla Nechiporuk[2,3], Geoffrey Shouse[1], Olga V. Danilova[1], Daniel Bottomly[2,4], Shannon K. McWeeney[2,4,5], Jeffrey W. Tyner[2,3,6], Stephen E. Kurtz[2,3], Alexey V. Danilov[1,2]

* - E.T. and T.L. contributed equally to this work

-SECURITY/CONFIDENTIALITY WARNING-

This message and any attachments are intended solely for the individual or entity to which they are addressed. This communication may contain information that is privileged, confidential, or exempt from disclosure under applicable law (e.g., personal health information, research data, financial information). Because this e-mail has been sent without encryption, individuals other than the intended recipient may be able to view the information, forward it to others or tamper with the information without the knowledge or consent of the sender. If you are not the intended recipient, or the employee or person responsible for delivering the message to the intended recipient, any dissemination, distribution or copying of the communication is strictly prohibited. If you received the communication in error, please notify the sender immediately by replying to this message and deleting the message and any accompanying files from your system. If, due to the security risks, you do not wish to receive further communications via e-mail, please reply to this message and inform the sender that you do not wish to receive further e-mail from the sender. (LCP301)

Alexey Danilov MD PhD

From: Nur Bruss
Sent: Thursday, February 17, 2022 4:00 PM
To: Alexey Danilov MD PhD
Subject: Re: CDDIS-21-2513

Yes, I agree

Best,

Nur Bruss
MS3/RA - Danilov Lab
Oregon Health and Science University/City of Hope Lymphoma Center
Bruss@ohsu.edu

From: Alexey Danilov MD PhD <adanilov@coh.org>
Sent: Thursday, February 17, 2022 2:19 PM
To: Elana Thieme; Nur Bruss; Tingting Liu; Vi Lam; Xiao Wang
Subject: [EXTERNAL] CDDIS-21-2513

Dear co-authors,

Will you please respond to this e-mail "Yes, I agree" if you agree with the following change

In reference to manuscript CDDIS-21-2513 **Dual BTK/SYK inhibition with CG-806 (luxeptinib) disrupts B-cell receptor and Bcl-2 signaling networks in mantle cell lymphoma** which has been provisionally accepted by Cell Death and Disease. Following revisions, we are planning to modify authorship:

1. Tingting Liu is co-first author
2. Carly Roleder is added to the author list

As a result, the change is as follows:

ORIGINAL author list:

Elana Thieme[1], Nur Bruss[2], Vi Lam[1], Xiaoguang Wang[1], Tamilla Nechiporuk[2,3], Geoffrey Shouse[1], Olga V. Danilova[1], Daniel Bottomly[2,4], Shannon K. McWeeney[2,4,5], Jeffrey W. Tyner[2,3,6], Stephen E. Kurtz[2,3], Alexey V. Danilov[1,2]* and Tingting Liu[1]*

* - A.V.D. and T.L. contributed equally to this work

UPDATED author list:

Elana Thieme[1*], Tingting Liu[1]*, Nur Bruss[2], Carly Roleder[1], Vi Lam[1], Xiaoguang Wang[1], Tamilla Nechiporuk[2,3], Geoffrey Shouse[1], Olga V. Danilova[1], Daniel Bottomly[2,4], Shannon K. McWeeney[2,4,5], Jeffrey W. Tyner[2,3,6], Stephen E. Kurtz[2,3], Alexey V. Danilov[1,2]

* - E.T. and T.L. contributed equally to this work

-SECURITY/CONFIDENTIALITY WARNING-

This message and any attachments are intended solely for the individual or entity to which they are addressed. This communication may contain information that is privileged, confidential, or exempt from disclosure under applicable law (e.g., personal health information, research data, financial information). Because this e-mail has been sent without encryption, individuals other than the intended recipient may be able to view the information, forward it to others or tamper with the information without the knowledge or consent of the sender. If you are not the intended recipient, or the employee or person responsible for delivering the message to the intended recipient, any dissemination, distribution or copying of the communication is strictly prohibited. If you received the communication in error, please notify the sender immediately by replying to this message and deleting the message and any accompanying files from your system. If, due to the security risks, you do not wish to receive further communications via e-mail, please reply to this message and inform the sender that you do not wish to receive further e-mail from the sender. (LCP301)

Alexey Danilov MD PhD

**From:**	Olga Danilova MD
**Sent:**	Thursday, February 17, 2022 4:53 PM
**To:**	Alexey Danilov MD PhD; Geoffrey Shouse DO PhD
**Subject:**	RE: CDDIS-21-2513

Yes, I agree.

Olga Danilova

From: Alexey Danilov MD PhD <adanilov@coh.org>
Sent: Thursday, February 17, 2022 3:00 PM
To: Geoffrey Shouse DO PhD <gshouse@coh.org>; Olga Danilova MD <odanilova@coh.org>
Subject: CDDIS-21-2513

Dear co-authors,

Will you please respond to this e-mail "Yes, I agree" if you agree with the following change

In reference to manuscript CDDIS-21-2513 **Dual BTK/SYK inhibition with CG-806 (luxeptinib) disrupts B-cell receptor and Bcl-2 signaling networks in mantle cell lymphoma** which has been provisionally accepted by Cell Death and Disease. Following revisions, we are planning to modify authorship:

1) Tingting Liu is co-first author
2) Carly Roleder is added to the author list

As a result, the change is as follows:

ORIGINAL author list:

Elana Thieme[1], Nur Bruss[2], Vi Lam[1], Xiaoguang Wang[1], Tamilla Nechiporuk[2,3], Geoffrey Shouse[1], Olga V. Danilova[1], Daniel Bottomly[2,4], Shannon K. McWeeney[2,4,5], Jeffrey W. Tyner[2,3,6], Stephen E. Kurtz[2,3], Alexey V. Danilov[1,2]* and Tingting Liu[1]*

* - A.V.D. and T.L. contributed equally to this work

UPDATED author list:

Elana Thieme[1*], Tingting Liu[1]*, Nur Bruss[2], Carly Roleder[1], Vi Lam[1], Xiaoguang Wang[1], Tamilla Nechiporuk[2,3], Geoffrey Shouse[1], Olga V. Danilova[1], Daniel Bottomly[2,4], Shannon K. McWeeney[2,4,5], Jeffrey W. Tyner[2,3,6], Stephen E. Kurtz[2,3], Alexey V. Danilov[1,2]

* - E.T. and T.L. contributed equally to this work

Alexey Danilov MD PhD

**From:**	Tamilla Nechiporuk <nechipot@ohsu.edu>
**Sent:**	Friday, February 18, 2022 8:05 AM
**To:**	Alexey Danilov MD PhD; Jeffrey Tyner; Steve Kurtz; Shannon McWeeney; Daniel Bottomly
**Subject:**	Re: CDDIS-21-2513

Yes I agree, thank you

From: Alexey Danilov MD PhD <adanilov@coh.org>
Sent: Thursday, February 17, 2022 2:59:08 PM
To: Jeffrey Tyner; Steve Kurtz; Shannon McWeeney; Daniel Bottomly; Tamilla Nechiporuk
Subject: [EXTERNAL] CDDIS-21-2513

Dear co-authors,

Will you please respond to this e-mail "Yes, I agree" if you agree with the following change

In reference to manuscript CDDIS-21-2513 **Dual BTK/SYK inhibition with CG-806 (luxeptinib) disrupts B-cell receptor and Bcl-2 signaling networks in mantle cell lymphoma** which has been provisionally accepted by Cell Death and Disease. Following revisions, we are planning to modify authorship:

1) Tingting Liu is co-first author
2) Carly Roleder is added to the author list

As a result, the change is as follows:

ORIGINAL author list:

Elana Thieme[1], Nur Bruss[2], Vi Lam[1], Xiaoguang Wang[1], Tamilla Nechiporuk[2,3], Geoffrey Shouse[1], Olga V. Danilova[1], Daniel Bottomly[2,4], Shannon K. McWeeney[2,4,5], Jeffrey W. Tyner[2,3,6], Stephen E. Kurtz[2,3], Alexey V. Danilov[1,2]* and Tingting Liu[1]*

* - A.V.D. and T.L. contributed equally to this work

UPDATED author list:

Elana Thieme[1*], Tingting Liu[1]*, Nur Bruss[2], Carly Roleder[1], Vi Lam[1], Xiaoguang Wang[1], Tamilla Nechiporuk[2,3], Geoffrey Shouse[1], Olga V. Danilova[1], Daniel Bottomly[2,4], Shannon K. McWeeney[2,4,5], Jeffrey W. Tyner[2,3,6], Stephen E. Kurtz[2,3], Alexey V. Danilov[1,2]

* - E.T. and T.L. contributed equally to this work

-SECURITY/CONFIDENTIALITY WARNING-

This message and any attachments are intended solely for the individual or entity to which they are addressed. This communication may contain information that is privileged, confidential, or exempt from disclosure under applicable law (e.g., personal health information, research data, financial information). Because this e-mail has been sent without encryption, individuals other than the intended recipient may be able to view the information, forward it to others or tamper with the information without the knowledge or consent of the sender. If you are not the intended recipient, or the employee or person responsible for delivering the message to the intended recipient, any dissemination, distribution or copying of the communication is strictly prohibited. If you received the communication in error, please notify the sender immediately by replying to this message and deleting the message and any accompanying files from your system. If, due to the security risks, you do not wish to receive further communications via e-mail, please reply to this message and inform the sender that you do not wish to receive further e-mail from the sender. (LCP301)
